# Protein structure prediction with in-cell photo-crosslinking mass spectrometry and deep learning

**DOI:** 10.1038/s41587-023-01704-z

**Published:** 2023-03-20

**Authors:** Kolja Stahl, Andrea Graziadei, Therese Dau, Oliver Brock, Juri Rappsilber

**Affiliations:** 1 Robotics and Biology Laboratory, Technische Universität Berlin, Berlin, Germany; 2 Technische Universität Berlin, Chair of Bioanalytics, Berlin, Germany; 3 Science of Intelligence, Research Cluster of Excellence, Berlin, Germany; 4 Si-M/‘Der Simulierte Mensch’, a Science Framework of Technische Universität Berlin and Charité - Universitätsmedizin Berlin, Berlin, Germany; 5 Wellcome Centre for Cell Biology, University of Edinburgh, Edinburgh, UK; 6 Present Address: Fritz Lipmann Institute, Leibniz Institute on Aging, Jena, Germany

**Keywords:** Molecular modelling, Proteomic analysis, Protein folding

## Abstract

While AlphaFold2 can predict accurate protein structures from the primary sequence, challenges remain for proteins that undergo conformational changes or for which few homologous sequences are known. Here we introduce AlphaLink, a modified version of the AlphaFold2 algorithm that incorporates experimental distance restraint information into its network architecture. By employing sparse experimental contacts as anchor points, AlphaLink improves on the performance of AlphaFold2 in predicting challenging targets. We confirm this experimentally by using the noncanonical amino acid photo-leucine to obtain information on residue–residue contacts inside cells by crosslinking mass spectrometry. The program can predict distinct conformations of proteins on the basis of the distance restraints provided, demonstrating the value of experimental data in driving protein structure prediction. The noise-tolerant framework for integrating data in protein structure prediction presented here opens a path to accurate characterization of protein structures from in-cell data.

## Abstract (web summary)

Limitations of Alphafold2 structure prediction are addressed by including experimentally determined distance constraints.

## Main

AlphaFold2 has shown unprecedented performance in CASP14, the Critical Assessment of protein Structure Prediction^1–3^, predicting two-thirds of the CASP targets with an approximately 1 Å root-mean-square deviation (r.m.s.d.) from the native backbone path^4^. This success, together with the reliable metrics provided by AlphaFold2 regarding the predicted accuracy of its models, is a tremendous achievement whose impact on life sciences is still unfolding.

AlphaFold2 predicts static models based on static input data. AlphaFold2 was trained on two information sources, the protein structures in the Protein Data Bank (PDB) and multiple sequence alignments (MSAs). This approach is challenged by targets that have insufficient evolutionary information, generating less confident or erroneous predictions^3^. For some classes of proteins, such as viral proteins, proteins from understudied organisms, antibodies^5^ and synthetic proteins, but also clinically relevant mutations^6^, evolutionary information may be misleading. Moreover, the x-ray structures underlying the model poorly reflect structural flexibility, multiple conformations and dynamic interactions. Structural restraints observed on proteins in solution, ideally in the cell, could help resolve these problems. Adding such restraints to the AlphaFold2 framework may then steer the prediction towards structural states occurring in situ under specific conditions.

Crosslinking mass spectrometry (MS) is capable of providing distance restraints that can be used in protein structure prediction^7–9^. In particular, photo amino acids (photo-AA) are readily incorporated by both prokaryotic and eukaryotic cells^10–12^, which opens up the possibility of probing the in situ conformation of proteins. Unlike most soluble crosslinkers, where data can be polluted by rare protein states, photochemistry accurately represents in-solution ensembles^13,14^. Furthermore, photo-AA crosslinks yield comparably tight distance restraints that align well with co-evolutionary contacts, which are the basis of most protein structure prediction methods, including AlphaFold2. They are in theory capable of ‘zero length’ crosslinking from the side chain to any heavy atom via a reactive carbene^10^ or alkyl diazo^15^ intermediate. Photo-leucine (photo-L) was used in mapping conformations and binders in purified systems^11,12^ but has not been used so far for in situ structure analyses. In general, the incorporation of amino acid analogs into the proteome is advantageous for crosslinking studies, because they allow the introduction of genetically encoded chemical entities that can react chemo-selectively at known locations in proteins^16^.

In this Article, we introduce AlphaLink, a structure prediction method that integrates experimental data from photo-AA crosslinking directly into the AlphaFold2 architecture. AlphaLink uses deep learning to merge co-evolutionary relationships and crosslinking data in distance space, exploiting the complementary nature of the data. We demonstrate that AlphaLink can leverage noisy experimental contacts to improve predictions of challenging targets on both simulated and real experimental data, steering predictions towards the in situ conformation of proteins. To test AlphaLink, we perform a large-scale crosslinking MS study with photo-L, identifying 615 in situ residue–residue contacts in *Escherichia coli* membrane fractions, unlocking the power of photo-AA in mapping proximal residues directly in cells. We show that even sparse crosslinking MS data can anchor predictions to particular conformational states, opening up the possibility of probing dynamics by hybrid experimental/deep learning approaches. We further extend AlphaLink to arbitrary distance restraints by introducing a second representation that encodes distance restraints as distograms^3^.

## Results

### AlphaLink: integrating crosslinks into AlphaFold2 via OpenFold

**Fig. 1:**
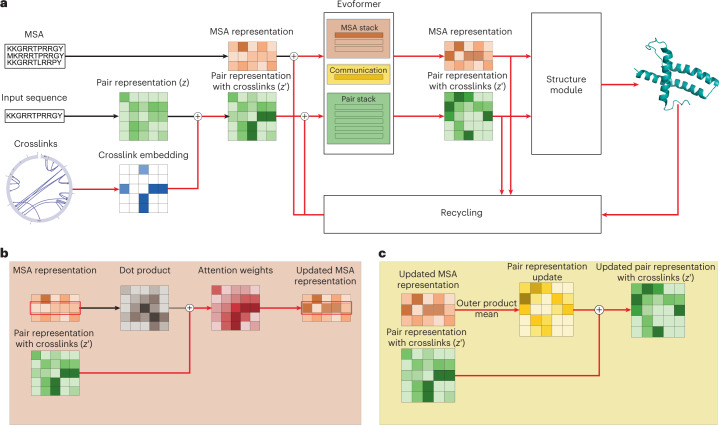
Information flow in AlphaLink. **a**, Overview of the information flow in AlphaLink. Crosslinks (blue) are embedded and added onto the pair representation (green). Impact of crosslinks shown in red. **b**, Crosslinks influence the retrieval of co-evolutionary information. They are used as a bias in the MSATransformer. **c**, The pair representation is updated with information from the MSAs that have been biased with the crosslinks.

Crosslinking MS data have been used to guide candidate selection for AlphaFold-multimer in protein–protein interaction studies and validate models^17,18^. To fully leverage the potential of crosslinking MS data in protein structure prediction, we develop AlphaLink, a framework incorporating crosslinks directly into OpenFold^19^. OpenFold is a trainable reproduction of AlphaFold2. The creators of OpenFold verified that the implementation produces identical results. OpenFold primarily exploits co-evolutionary relationships. The main difficulty in merging multiple information sources is to find a suitable representation that facilitates integration and at the same time avoids information loss. OpenFold operates both in distance space (Evoformer) and in 3D space (Structure Module). Photo-AA crosslinking MS data provide distance restraints that naturally fit into the distance space of OpenFold, since they yield similar distances to co-evolutionary contacts by directly linking amino acids via diazirine chemistry. Co-evolutionary relationships and photo-AA crosslinks provide complementary and corroborating information. The sparsity of crosslinks can be compensated with co-evolutionary information. Accurate crosslinking data can act as an anchor in these cases. AlphaLink exploits this relationship by merging crosslinking MS and co-evolutionary data via the Evoformer, injecting crosslinks into the pair representation (*z*), yielding a consistent and unified constraint set (Fig. 1).

We introduce two representations to encode crosslinking information. The experimental data are represented as either soft labels or distance distributions (distograms). In the case of soft labels, each contact is weighted by the link-level false discovery rate (FDR) of the dataset (1-FDR) or, if present, the per-restraint FDR to indicate confidence in crosslink assignment. Distograms allow us to generalize to arbitrary distance restraints. A particular crosslinker (or distance restraint in general) is represented by a distance distribution. Contact-like restraints can be represented by uniformly distributed distograms for the given cutoff. We model uncertainty directly in the representation by adjusting the probability mass according to the FDR. The distogram is designed to match the distogram that is predicted by the Evoformer from the pair representation that consists of 64 bins. We use the same binning for the first 64 bins and extend the distogram further to 128 bins, spanning from 2.3125 Å to 42 Å.

We embed the restraints and add them to the pair representation of OpenFold, which is later mapped into 3D space (Fig. 1a). The embedding is similar to the recycling embedding in AlphaFold2. The Evoformer jointly updates the MSA and the pair representation. The MSA transformer (Fig. 1b) retrieves co-evolutionary information and updates the MSA representation. The retrieval is biased with the pair representation that includes the experimental crosslinking information supplied by the user. The outer product mean (Fig. 1c) in turn updates the pair representation. This coupling maximizes synergy between MSA and experimental information and allows the network to perform noise rejection, that is, the rejection of misassigned experimental or co-evolutionary relationships or of contacts that do not support other strands of information leading to a consensus model.

We initialized OpenFold with the original weights of AlphaFold2 and fine-tuned the network with the newly added crosslinking bias. We followed the refinement training regime outlined in the AlphaFold2 paper, except that we subsampled the number of effective sequences (*N*_eff_) to simulate challenging targets. In light of the limited availability of experimental crosslink data for training, we simulated photo-crosslinking MS data (Methods) that included simulated experimental noise in the form of false residue–residue contacts at the given FDR.

### Integrating photo-AA crosslinks enables noise-tolerant prediction of challenging targets

**Fig. 2:**
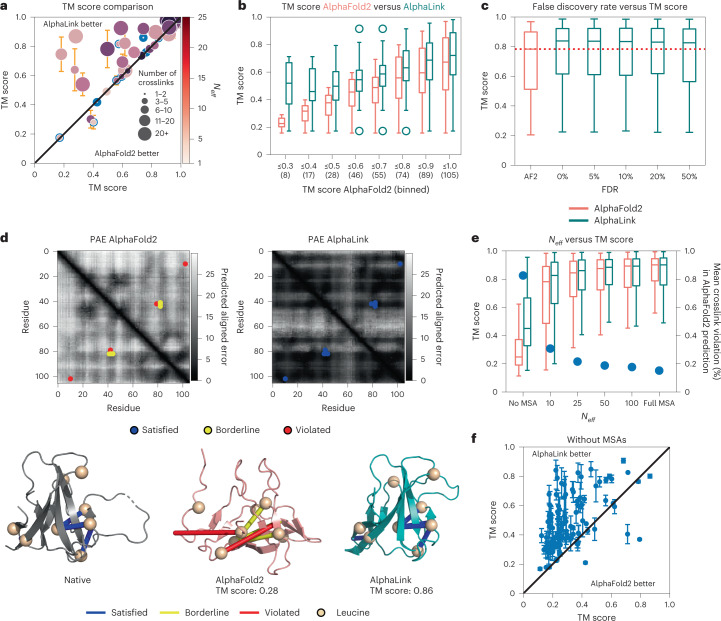
AlphaLink performance comparison against AlphaFold2. **a**, TM score comparison on 49 CAMEO targets with *N*_eff_ ≤ 25. Error bars represent the 95% confidence intervals (*N* = 10). Points show the mean. TM score improves on average by 19.2%. **b**, Performance on 60 CASP14 and 45 CAMEO targets broken down by TM score (*N*_eff_ = 10). AlphaLink improves on average by 15.2%. Number of targets in each range bin in brackets below. **c**, Performance on 60 CASP14 targets (*N*_eff_ = 10) with different noise levels (FDR 0%, 5%, 10%, 20% and 50%). AlphaLink improves in the median for all noise levels. Performance shows robust noise rejection. Dotted line shows median performance of AlphaFold2. **d**, Predicted aligned error of AlphaFold2 (left) and AlphaLink (right) on T1064 with *N*_eff_ = 10 (top) and predicted structures (bottom). Light regions signify high uncertainty. Sparse restraints decrease uncertainty across the whole protein. Satisfied crosslinks <10 Å Cα–Cα highlighted in blue, borderline crosslinks (10–15 Å Cα–Cα) in yellow, and violated crosslinks >15 Å Cα–Cα in red. Possible crosslinking sites (leucines) are shown as spheres. Regions with violated crosslinks in the AlphaFold2 prediction (left) increase in certainty (darker regions). TM score improves from 0.28 to 0.86. **e**, Performance on 60 CASP14 targets (*N*_eff_ = 10) as a function of MSA size (*N* = 100, 10 MSAs and 10 crosslink sets). Dots represent the mean percentage of nonsatisfied crosslinks (>10 Å Cα–Cα) in the AlphaFold2 prediction. Improvement on average for all but full MSA size. Crosslink violation decreases and crosslink utility diminishes with increasing MSA size. Largest utility for *N*_eff_ < 25. **f**, Performance without MSAs on 60 CASP14 and 45 CAMEO targets. AlphaLink predicts the correct fold (TM score >0.5) for 43/105 (13/105 for AlphaFold2). Error bars represent the 95% confidence interval (*N* = 10). Points show the mean. In all box plots, the line shows the median and the whiskers represent the 1.5× interquartile range. Source data

**Extended Data Fig. 1:**
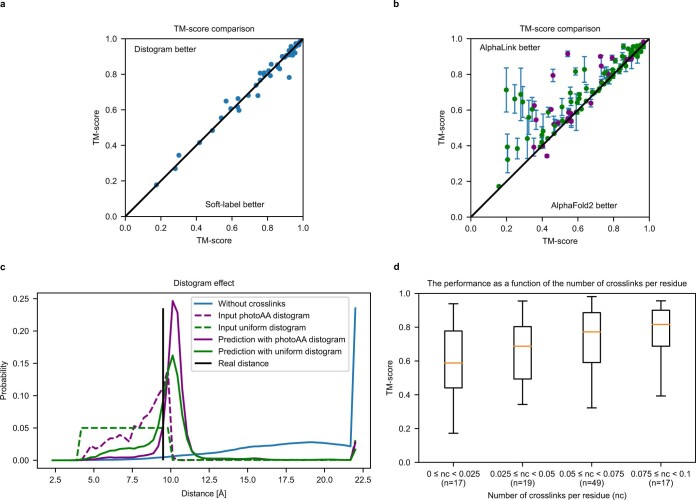
Performance on benchmark data set and distogram effect. a- TM-score comparison (*N* = 10) 49 challenging CAMEO targets for the network trained with the soft-label representation and the network trained with the distogram representation. Each target was predicted with 10 randomly subsampled crosslink sets. Scatter points show the mean. The photo-L crosslinks were represented as a uniformly distributed distogram. The performance is on par. b- Performance on 60 CASP14 and 45 CAMEO (*N*_eff_ = 10). AlphaLink improves the TM-score on average by 15.2%. The error bars represent the 95% confidence interval (*N* = 10). Points show the mean. Purple highlights multi-domain targets, green highlights single-domain targets. c- The effect two different distogram inputs (dashed) have on the distogram AlphaLink predicts (solid) between residues 11 and 103 for T0164. The solid black line designates the real distance. Blue shows the distogram without using crosslinks. The green distogram mimics an upper bound contact restraint, while the purple distogram is the expected distance distribution for simulated photo-L and photo-K crosslinks on the training set. Predicted distogram based on the expected photoAA distance distribution (purple, solid) is more narrow compared to the prediction with the uniform distance distribution (green, solid). Using a distance distribution improves the prediction from TM-score of 0.68 to 0.7. We show the first 64 bins of the input distograms and sum up the probabilities for the rest. In the absence of this restraint, the prediction has a TM-score of 0.28 (*N*_eff_ = 10). d- Performance of AlphaLink as a function of the number of crosslinks per residue (*N*_eff_ = 10). 2 bins (nc > 0.1) were omitted because they only contained 1 and 2 samples. Performance generally increases with more crosslinks per residue. The line shows the median and the whiskers represent the 1.5x interquartile range. Source data

We tested AlphaLink on 49 challenging CAMEO targets (*N*_eff_ ≤ 25, no MSA subsampling, Supplementary Data 1) (Fig. 2a). AlphaLink outperforms AlphaFold2, substantially improving the performance on targets with more than 20 crosslinks. Integrating simulated photo-L data improves the TM score on average by 19.2 ± 16.3% (95% confidence interval) (Fig. 2a). Encoding the crosslinks as distograms instead performs virtually the same (Extended Data Fig. 1a).

We further curated a second benchmark dataset consisting of 60 CASP14 targets and 45 CAMEO targets (Supplementary Data 1). To simulate challenging targets and to control for the MSA influence, we subsampled the MSAs to *N*_eff_ = 10 and ignored structural templates. Here AlphaLink improves the TM score on average by 15.2% (Extended Data Fig. 1b). For particularly challenging targets (*N* = 28), where AlphaFold2 fails to predict the correct fold (TM score ≤0.5), the TM score improves on average by 50.6% (Fig. 2b). AlphaLink predicts the correct fold (TM score >0.5) of 14 of these. We tested the noise rejection capabilities of AlphaLink on 60 CASP14 targets by adding false links to simulate multiple noise levels. The performance is roughly constant with 10%, 20% or 50% false links (Fig. 2c) and still outperforms AlphaFold2, demonstrating AlphaLinks’ robustness to different noise levels. Overall, the method achieves a crosslink satisfaction (<10 Å Cα–Cα) on average of 85 ± 1.2% (95% confidence interval) after three recycling iterations, and 88.3 ± 1.2% (95% confidence interval) of the simulated crosslinks with <10 Å Cα–Cα in the crystal structure are satisfied.

The sparse crosslink data act as anchor points that serve to pull the entire prediction towards the right solution (Fig. 2d). For CASP target T1064 (*N*_eff_ = 10), four crosslinking restraints are sufficient to both drive the prediction to the native state (TM score improves from 0.28 to 0.86) and to decrease the predicted aligned error across the whole protein, including areas not covered by the crosslinking data. The crosslinking information has a wide-ranging impact due to its combination with the co-evolutionary and structural information embedded in the pair representation, which is used as a bias to retrieve contacts consistent with experimental data. Effectively, this improves the efficiency of using co-evolutionary information in AlphaFold2. Extended Data Fig. 1c shows the effect of using different distograms to encode a restraint between residues 11 and 103 in T1064. The Evoformer predicts a narrower distogram when using the expected distance distribution of photo-AA crosslinks as a prior, when compared with the uniform prior of an upper bound distance restraint. This representation slightly improves the prediction (TM score 0.68 to 0.7). The performance as a function of the number of crosslinks per residue is shown in Extended Data Fig. 1d. The performance generally increases with an increase in the number of crosslinks per residue. The main advantage of the distogram representation is enabling the user to inject distance restraints from different crosslinkers or even different experimental approaches into AlphaLink.

We test the performance of AlphaLink at different *N*_eff_ levels to investigate the effect of crosslinks on targets with varying difficulty (Fig. 2e). The performance of both AlphaFold2 and AlphaLink deteriorates in absence of sufficiently large MSAs (Fig. 2e). Crosslinks can compensate for smaller MSA sizes. In fact, photo-AA crosslinks alone without any MSA information allow us to predict the correct fold (TM score >0.5) of 43/105 benchmark targets, compared with 13/105 for AF2 without MSA information. The mean improvement in TM score increases to 75 ± 13.5% (95% confidence interval) over all targets (Fig. 2f). The benefit of crosslinks slowly disappears with a *N*_eff_ > 50. This is at least partly due to the fact that most crosslinks are already satisfied when predicting with full MSAs (Fig. 2e). Rather than finding any solution that fits the crosslinks, our network appropriately weighs crosslinking MS information against the MSAs and uses it to guide the prediction to a more accurate solution. Note that as MSA size increases, the network will rely more on MSA information than on crosslinks—hence, we implement settings with different MSA subsamplings in the AlphaLink software package.

In summary, AlphaLink enables users to use sparse distance restraints to bias AlphaFold2 predictions, robustly handling noise, directly at the inference stage, due to their synergistic implementation in the network design.

### Photo-L as an in situ structural probe

**Fig. 3:**
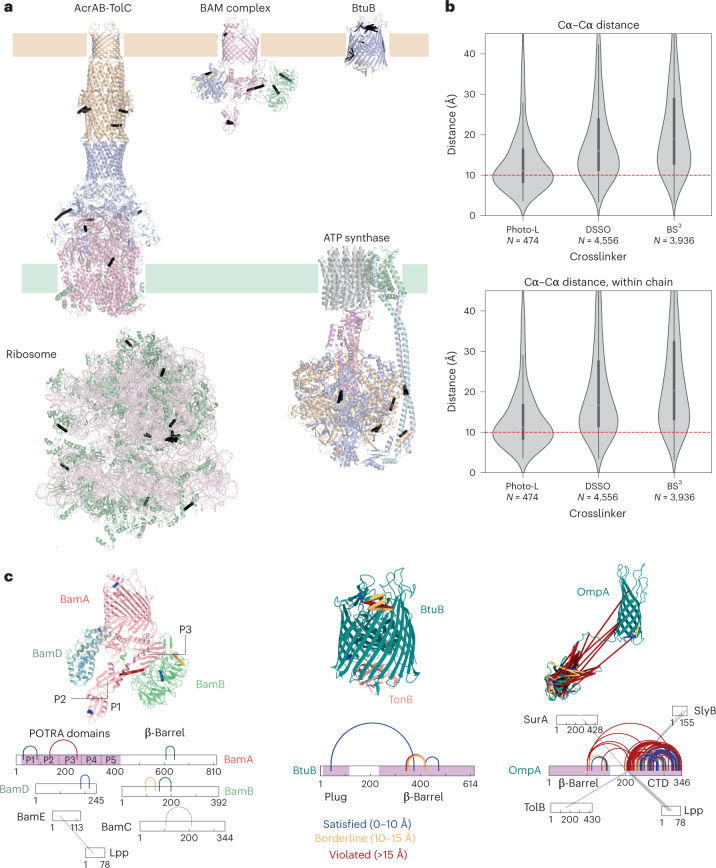
In situ photo-L crosslinking MS in *E. coli*. **a**, Distance restraints from in-cell photo-L crosslinking MS mapped onto cellular complexes. **b**, Distance distributions for photo-L crosslinks mapped onto known structures, taking only a single conformer per protein. Bissulfosuccinimidyl suberate (BS^3^) and disuccinimidyl sulfoxide (DSSO) distograms are obtained by mapping the crosslinks of Lenz et al. ^33^. The distograms are derived by accounting for homo-multimers (top) or mapping to only within-chain distances (bottom). **c**, Distance restraint analysis of outer membrane proteins crosslinked with photo-L. The dot represents the median, and the whiskers represent the 1.5× interquartile range. Source data

**Extended Data Fig. 2:**
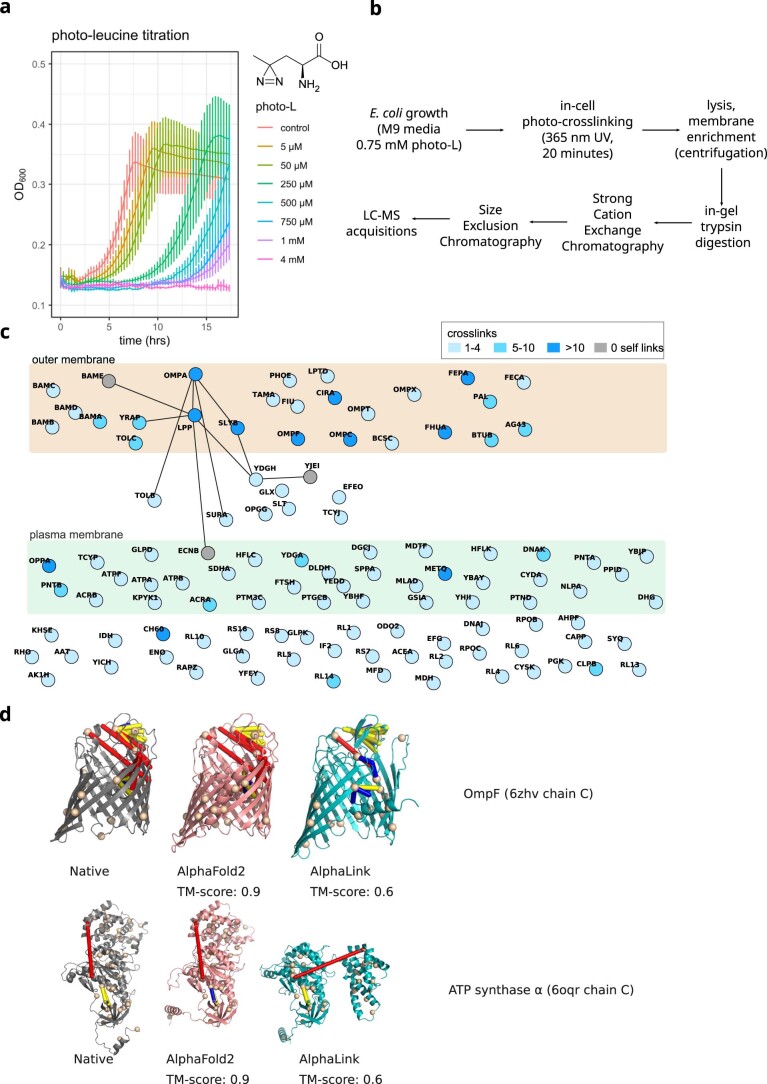
Photo amino acid crosslinking MS workflow and membrane outliers. a- E. coli K12 grown with increasing concentration of photo-L in M9 minimal media and structure of photo-L. Average cell density with standard error across 3 individual colonies is plotted. b- Workflow for two-dimensional fractionation of peptides from E. coli crosslinked with photo-L in cell. Error bars representing standard error. c- Nodes represent proteins detected with at least one crosslink, and edges indicate the presence of at least a crosslinked residue pair between proteins. Nodes are coloured according to the number of crosslinks detected in that protein. d- Two examples where crosslinks negatively impact the performance in AlphaLink. The crosslink sets contain overlength links (shown in red in the native structure) which cause movements in AlphaLink (for example, domain movement in ATP synthase - likely a false positive link). OmpF is homo-multimeric, the overlength links might stem from links between different subunits. Source data

To generate a large-scale experimental photo-AA dataset required for testing such an application, we derived in situ structural restraints on the *E. coli* membrane fraction by crosslinking MS of cells grown on photo-L-containing medium. We optimized the growth protocol to maximize incorporation while maintaining a low level of cytotoxicity (750 μM photo-L in the medium, Extended Data Fig. 2a), ultraviolet (UV) illuminated the cells for crosslinking and then enriched the cell membrane of the crosslinked cells. The proteins were digested, and the resulting peptides subjected to two-dimensional fractionation, combining strong cation exchange and size exclusion chromatography (Extended Data Fig. 2b). Mass spectrometric analysis then led to the identification of 615 residue pairs involving 112 proteins at 5% link-level FDR (Fig. 3a, Extended Data Fig. 2c and Supplementary Data 2 and 3). Several crosslinks are detected among β-barrel proteins and proteins in the intermembrane space, including porins and known membrane complexes (Fig. 3a and Extended Data Fig. 2a). When visualized on known protein structures, the experimental crosslinks provide a median distance of 11.1 ± 8.1 Å Cα–Cα (mean ± standard deviation) (Fig. 3b), indicating the contact-like nature of these crosslinks in line with their implementation in AlphaLink. This is further supported by the fact that we exclude crosslinks within the same tryptic peptide, and between consecutive peptides in our analysis.

Photo-L provides validation for the in situ conformation of multiprotein complexes such as the AcrAB-TolC multidrug efflux pump, ribosome and ATP synthase (Fig. 3a). The crosslinks are consistent with previously characterized conformations of the bacterial outer membrane barrel assembly machinery (Bam). However, a link between the P2 and P3 domains highlights the flexibility of these modules (Fig. 3c), which are known to undergo large structural rearrangements in outer membrane protein folding and insertion. A total of 153 crosslinks are detected for the highly abundant protein OmpA. OmpA is made up of a β-barrel connected via a 20-residue linker to a C-terminal domain. It is also known to oligomerize in vivo, and this interaction is thought to be mediated by the C-terminal domain. The crosslinks between the β-barrel, linker and C-terminal domain highlight the relative flexibility of these modules (Fig. 3c) and point to potential contacts made between multiple copies of the C-terminal domain. In several plug-containing β-barrel proteins, such as FhuA and BtuB, photo-L links the position of the central plug with the membrane barrel in a way that is consistent with previous structures (Fig. 3c), validating the arrangement of these two modules in the functional cycle of the proteins. These crosslinks highlight the potential of photo-L to provide in situ residue–residue contacts regardless of solvent accessibility, providing insight into function for critical domain contacts.

### Structure prediction with in situ photo-L data

**Fig. 4:**
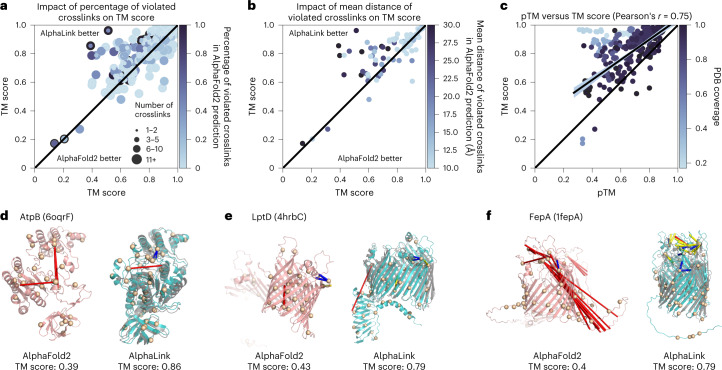
Structure prediction with in-cell photo-L crosslinking MS data of the *E. coli* membrane fraction. **a**, Comparison of TM score with annotated number of links (marker sizes) and percentage of nonsatisfied (>10 Å) crosslinks (color gradient) in the AlphaFold2 prediction. Performance improvement is bigger for targets with a higher percentage of nonsatisfied crosslinks in the base prediction (darker circles). Each target is predicted ten times with different MSA subsamples at *N*_eff_ = 10. AlphaLink outperforms AlphaFold2 on average. **b**, Comparison of TM score with annotated mean distance of nonsatisfied crosslinks in the base AlphaFold2 prediction (color gradient). Prediction quality improves with stronger crosslink violations (darker circles). **c**, We show the calibration of the pTM. On predictions that are at least 80% covered by the crystal structure, the correlation is 0.75. The true TM score is generally underestimated, meaning that the pTM score of AlphaLink is a conservative estimate. The shaded area corresponds to the 95% confidence interval. Line shows the linear fit. **d**, Prediction of the ATP synthase subunit AtpB by AlphaFold2 and AlphaLink using in-cell photo-L crosslinks at *N*_eff_ = 10. **e**, Prediction of the outer membrane lipopolysaccharide assembly protein. **f**, Prediction of the ferrienterobactin receptor. In all three cases, the in-cell crosslinking data helps AlphaLink position different regions of the protein relative to each other, yielding a performance improvement over AlphaFold2. The crystal structure of the target protein is shown in gray, overlaid with the AlphaLink prediction. Source data

To test AlphaLink on experimental data, we predicted the proteins in the crosslinking MS dataset of the *E. coli* membrane fraction. We focused our evaluation on the 31 targets with high-resolution structures that had a median of five crosslinks (Fig. 4). Each target was predicted with ten randomly subsampled MSAs at *N*_eff_ = 10, yielding 310 predictions (Supplementary Data 4). We subsampled the MSAs to counter overfitting, because the targets were probably part of the AlphaFold2 training set. Even with *N*_eff_ = 10, 65% of the AlphaFold2 predictions exceed a TM score of 0.8. AlphaLink improves performance measured by TM score on average by 5.2 ± 1.9% (95% confidence interval) across all proteins relative to AlphaFold2.

On targets where AlphaFold2 does not provide accurate models (TM score <0.8), AlphaLink with experimental data improves the TM score on average by 15.9 ± 4.6% (95% confidence interval). The improvement increases to 47.8 ± 24.8% (95% confidence interval) for AlphaFold2 predictions below a TM score of 0.5. We predict the correct fold (TM score >0.5) for ten additional proteins. This shows that simulated crosslinking MS data successfully model the features of experimental photo-AA restraints. For the 204 AlphaFold2 predictions with a TM score of 0.8 or higher, the performance is unaffected. At high TM scores, side-chain conformations begin to play a role, and crosslinking MS data do not have the resolution necessary to improve side-chain predictions.

To better judge the utility of the crosslinks for a given target, we include the percentage of nonsatisfied crosslinks in the baseline AlphaFold2 prediction (Fig. 4a) and also consider the mean distance of the nonsatisfied crosslinks in the AlphaFold2 prediction (Fig. 4b). We set the cutoff for violated crosslinking restraints to 10 Å Cα–Cα in the crystal structure. Many targets are not completely covered by the crystal structure. Therefore, we can analyze only a subset of the crosslinks. Crosslinks that are already satisfied in the AlphaFold2 predictions do not contribute novel information. On average, there are 0.5 violated crosslinking restraints per prediction at a cutoff of 10 Å Cα–Cα. Indeed, the TM score improvement of AlphaLink generally increases wherever AlphaFold2 makes a prediction containing unsatisfied crosslinks. We further show that the predictions that improved the most have unsatisfied crosslinks with large distances in the baseline prediction (Fig. 4b). Here crosslinks add the most value, and for some predictions a single crosslink is enough to improve the quality considerably (TM score 0.39 to 0.86 for target AtpB). Extended Data Fig. 2d shows two examples where adding crosslinks negatively impacts the prediction quality. In the case of OmpF there are multiple overlength crosslinks (highlighted in red in the native structure) that might stem from crosslinking different subunits, since OmpF is a homo-multimer. For the ATP synthase α subunit there is one overlength crosslink that is probably a false positive. Here, although the link is rejected in the end, it still induces a domain movement that leads to a worse prediction.

**Extended Data Fig. 3:**
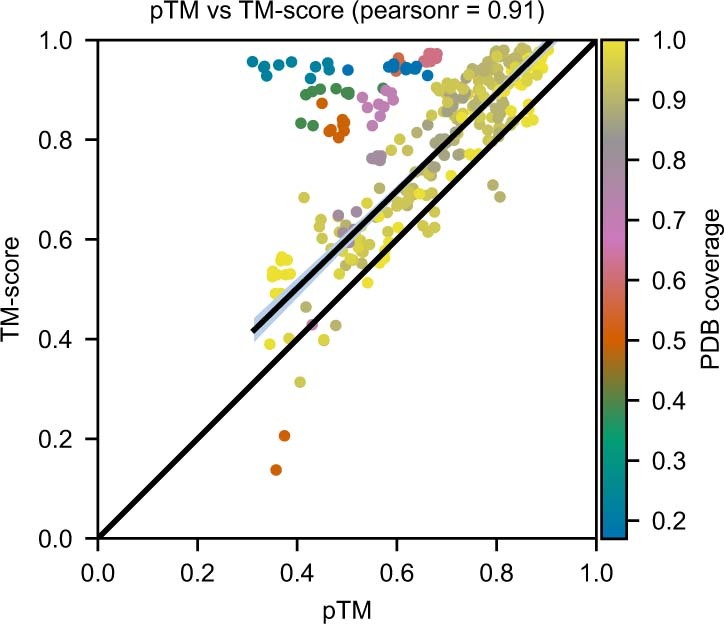
AlphaFold2 pTM correlation. Calibration of the predicted TM-score (pTM) on *N* = 320 predictions of the E. coli membrane fraction dataset. On predictions that are at least 80% covered by the crystal structure, the correlation is 0.91. The true TM-score is generally underestimated, meaning that the pTM-score of AlphaFold2 is a conservative estimate. The shaded area corresponds to the 95% confidence interval. Line shows the linear fit. Source data

To investigate the correlation between predicted and true TM score for the predictions of the membrane fraction, we compute the fit on the predictions where the crystal structure covers at least 80% of the protein (Fig. 4c). The Pearson correlation coefficient is 0.75. We generally underestimate the true TM score. The correlation is in line with the baseline AlphaFold2 model (Extended Data Fig. 3), indicating that model confidence estimates of AlphaLink are comparable to AlphaFold2, allowing for users to reliably interpret predictions.

**Extended Data Fig. 4:**
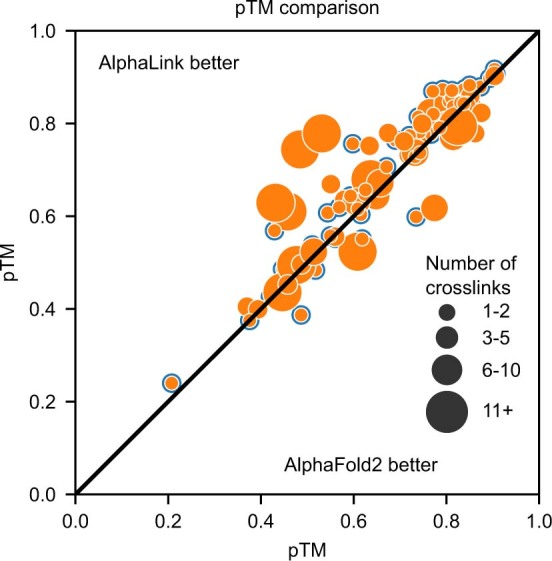
pTM comparison on 96 proteins from the E. coli membrane fraction dataset. pTM comparison on *N* = 96 proteins from the E. coli membrane fraction dataset. Each points is one protein with one MSA subsample (*N*_eff_ = 10). The higher pTM of AlphaLink indicates improved structures. Source data

Extended Data Fig. 4 shows the predicted TM score (pTM) on a total of 96 targets, which include proteins where no structure is available. Each protein was predicted with one randomly subsampled MSA (*N*_eff_ = 10). The pTM indicates possible improvements over AlphaFold2 on these structures as well.

### Probing conformational dynamics in situ

**Fig. 5:**
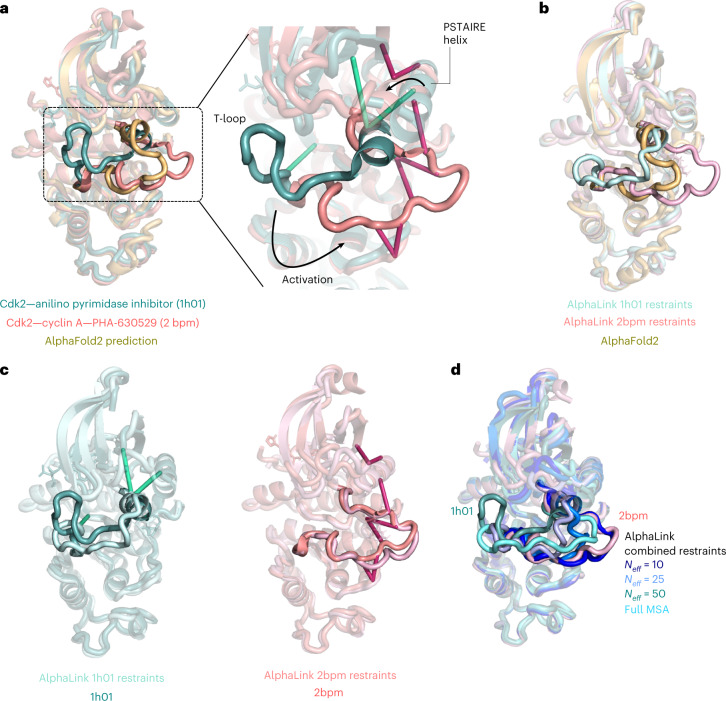
Photo-AA data guiding prediction of specific conformational states. **a**, Left: structures of the monomeric, inhibited conformation of Cdk2 (teal)^34^ and the cyclin A-activated conformation (salmon)^35^ overlaid with the AlphaFold2 prediction of Cdk2 performed at *N*_eff_ = 10. Right: focus on the T-loop and PSTAIRE helix involved in protein activation, with the two photo-AA restraint sets fed to AlphaLink colored according to the corresponding protein state. **b**, Comparison of the AlphaFold2 prediction with the two predictions of AlphaLink made with restraint sets corresponding to the active or inactive conformation of Cdk2, showing that the photo-AA data drive the prediction to either the active or inhibited conformation. **c**, Middle: overlay of the AlphaLink prediction with the crystal structure for the inhibited state. Right: overlay of the AlphaLink prediction with the structure for the cyclin A-bound state, showing the entire conformation of the loop is correctly predicted despite only sparse restraints being present. **d**, Outcome of predicting with a combined set of restraints. At low *N*_eff_ values, the crosslinks drive the prediction towards the cyclin E-bound state. As the MSA information increases, the prediction is steered more towards the inhibited state and closer to the AlphaFold2 prediction.

**Extended Data Fig. 5:**
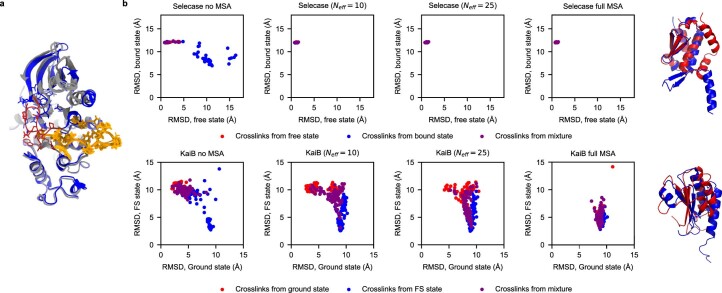
Predicting multiple conformations. a- Cdk2 predictions by AlphaFold2.2 with default settings (full MSA, 5 models predicted, 5 random seeds per model), in gray, overlaid with the structure of the inhibited conformation of Cdk2 (blue, pdb 1h01). The T-loop is highlighted in orange in the AlphaFold2 predictions and in red in the inhibited conformation structure. All 25 AlphaFold2 predictions converge on the cyclin A-bound conformation of the T-loop and PSTAIRE helix of Cdk2. b- Each points is one prediction with a randomly sampled crosslink (FDR = 5%) set which includes additional links from the highlighted conformation. 100 samples per conformation. For Selecase, AlphaLink always predicts the free state with high accuracy if we use MSAs. Without MSAs, some predictions are driven towards the bound state. For KaiB, AlphaLink is able to predict the ground state with good precision without MSAs. Introducing co-evolutionary information leads to better clustering, although some of the ground state predictions now end up in the average state. This movement proceeds with more co-evolutionary information. With full MSAs, almost all predictions have moved to the fold-switched state. Source data

To probe whether experimental distance restraints can act as anchors to drive predictions towards different energy minima in multistate proteins, we simulate a proof-of-concept experiment on the human cyclin-dependent protein kinase Cdk2, a drug target in cancer therapy^20^. Activation of Cdk2 in the S phase proceeds via a conformational change in the T-loop (residues 145–165) and the PSTAIRE helix (residues 45–55) triggered by binding of cyclin A^21^. There are several structures of Cdk2 in various states of activation^22,23^. If Cdk2 is predicted without structural templates with AlphaFold2 (*N*_eff_ = 10), the T-loop is predicted in an intermediate conformation between the apo, auto-inhibited state and the cyclin A-bound conformation (Fig. 5a). Presumably, the intermediate conformation of this loop in the AlphaFold2 prediction is a consequence of co-evolutionary information driving it towards both the open and the closed state. When run with full MSA information, all AlphaFold2 predictions converge to the cyclin A-bound state (Extended Data Fig. 5a), failing to predict the inactive conformation.

We simulate two photo-crosslinking MS experiments in which the protein was acquired in either its inhibited or in its cyclin A-bound states, generating two sets of sparse restraints for the T-loop (Supplementary Table 1). Such experiments may be carried out on the purified protein or in cells before protein purification. We then predict the Cdk2 structure using AlphaLink with these restraints, showing that the loop structure is driven towards the appropriate conformation (Fig. 5b). The crosslinks act as anchor points positioning the whole T-loop in the appropriate configuration for the cyclin A-bound state, with a Cα r.m.s.d. of 1.24 Å on residues 145–165 to PDB 2bpm (Fig. 5c). The same is true for the inactive state of the loop. In this case however, lack of leucine and lysine residues around T160 in the structure leads to a lack of sufficient restraints to capture the fully closed loop conformation, leading to a slightly higher Cα r.m.s.d. to the target structure (3.19 Å to 1h01), while still outperforming the AlphaFold2 prediction (Cα r.m.s.d. 6.29 Å to 1h01). This higher r.m.s.d. is also consistent with the fact that the T-loop is not rigid in its inhibited, dephosphorylated state, as highlighted by multiple crystal structures and molecular dynamics simulations^24^. AlphaLink successfully folds the short helix within the T-loop (residues 147–153) in the inactive state, and unfolds them into an extended conformation when given restraints for the cyclin A-bound state. It also correctly predicts the position and rotation of the PSTAIRE helix, despite having only two restraints in this region in the inactive conformation dataset, and three in the active dataset. In the case of a mixture of restraints, the prediction converges on the cyclin-A bound state at *N*_eff_ = 10 (Fig. 5d). This conformer is not produced by AlphaFold2. Increasing the MSA steers the prediction towards a middle ground that is more similar to the AlphaFold2 prediction. We interpret this as the algorithm performing noise rejection on a subset of crosslinks in the mixture and using the rest as anchor points to drive a prediction towards a particular solution.

To further show the influence of the MSA, we predict the conformation of the fold-switching protein KaiB (Extended Data Fig. 5b) with photo-L crosslinks simulated for the ground state, the fold-switched state or a mixture added on top of random sets of simulated photo-L crosslinks. At low *N*_eff_, AlphaLink predicts both conformers accurately when given unique sets of crosslinks, but as MSA evidence gets larger, the prediction converges to one state for both sets. This result reproduces the outcome of running AlphaFold on KaiB with different, clustered subsamples of the MSA^25^. Predictions with mixed crosslinks lead to different outcomes at different *N*_eff_ values, as observed in the case of Cdk2, pointing to the fact that crosslinking is weighted against the MSA depending on the information content and size of both strands of information. In multiple simulated crosslinking datasets for the protein selecase (Extended Data Fig. 5b), even without MSAs, most predictions end up in the conformation observed in the monomeric state of the protein state, although some predictions corresponding to the bound state are observed when given unique crosslinks in the absence of MSA information.

These results demonstrate that AlphaLink can be used to obtain high-quality predictions of particular conformations of proteins given sets of restraints obtained under different conditions, enabling direct monitoring of conformational states in solution and in situ.

## Discussion

**Extended Data Fig. 6:**
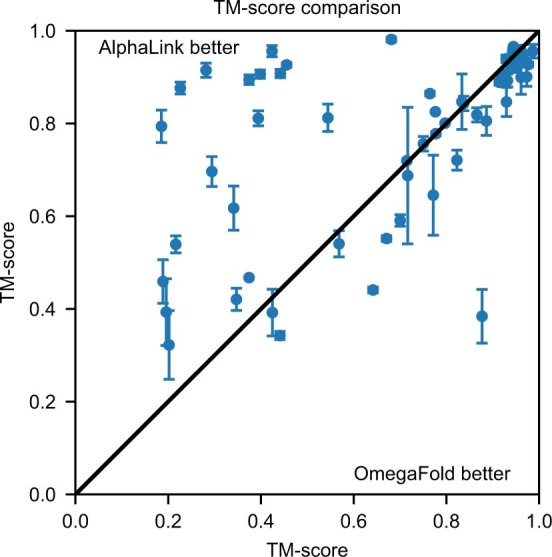
AlphaLink vs OmegaFold. AlphaLink vs OmegaFold performance on 58 CASP14 targets with *N*_eff_ = 10 (test set) with simulated photo-L crosslinks. AlphaLink improves the TM-score on average by 36.7% ± 76.6. AlphaLink improves 10 additional targets past a TM-score > 0.5. Shown here is the mean and 95% confidence interval (*N* = 10) for AlphaLink and the corresponding performance of OmegaFold. Source data

We presented AlphaLink, a method for integrating crosslinking MS restraints derived from photo-AA-labeled cells into AlphaFold2, via OpenFold. Merging photo-crosslinking MS and MSA information in a deep learning framework allows us to leverage their respective strengths and compensate for their weaknesses. Our approach uses the experimental data to bias the retrieval of evolutionary relationships by the Evoformer updating the pair representation. The iterative nature of the AlphaLink architecture leads to noise rejection and robustness to experimental error. Our implementation of experimental restraints also translates to other methods with similar architectures, such as OmegaFold^26^, which replace MSAs with protein language models (Extended Data Fig. 6).

**Extended Data Fig. 7:**
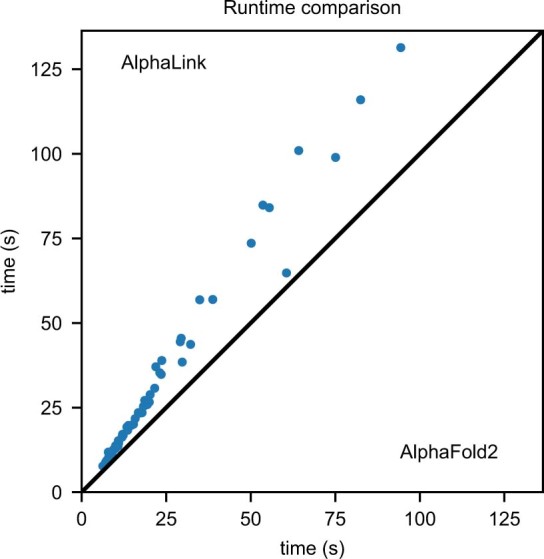
AlphaLink vs AlphaFold2 timings. Tested on *N* = 60 CASP14 targets with *N*_eff_ = 10. We ran inference, including relaxation on pre-computed features with model_5_ptm. Timed on a node with a single Nvidia A100 80GB GPU and 2 Intel XEON Gold 5118 CPUs (2 × 24 cores) with 2.3 GHz. Mean running time (s) for AlphaLink is 31.7 ± 7.4 s and 22.14 ± 5.16 s for AlphaFold2. Source data

The results in this study were achieved by refining the AlphaFold2 model parameters with simulated photo-AA data, as we were not able to fully retrain the OpenFold network to derive model parameters due to computational resource limitations. Nevertheless, the results demonstrate an improvement in prediction quality for challenging targets as a result of incorporating photo-AA restraints. The prediction times increase 1.4× compared with AlphaFold2 (Extended Data Fig. 7).

The information sources have different characteristics that match well. Crosslinking MS provides concrete distance information that can corroborate or refute amino acid associations picked up by co-evolution^7,27,28^. As such, crosslinking MS information has already been used to independently validate models from AlphaFold2 (refs. ^17,18^). Moreover, genetic code engineering allows the use of amino acid analogs to substitute for encoded amino acids in protein translation. Thus, leucine positions in the proteome can be replaced to varying extents by photo-L. This amino acid has been linked with the evolutionary development of tertiary folds^29^ and is usually found in the hydrophobic cores of proteins. Leucine crosslinking may therefore provide critical information that can guide fold prediction effectively.

**Extended Data Fig. 8:**
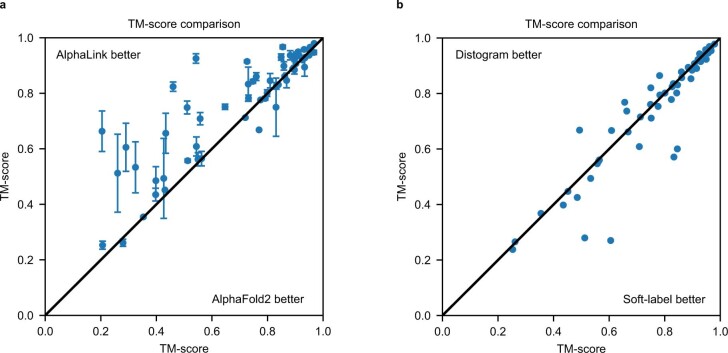
AlphaLink with soluble crosslinker on CASP14. a- AlphaLink vs AlphaFold2 performance on 59 CASP14 targets with *N*_eff_ = 10 (test set) with simulated SDA crosslinks. AlphaLink improves the TM-score on average by 16.4% ± 9.7. AlphaLink predicts 5 additional targets with a TM-score > 0.5. Shown here is the mean and 95% confidence interval (*N* = 10) for AlphaLink and the corresponding performance of AlphaFold2. b- We compare the mean TM-score (*N* = 10) per target on 59 CASP14 targets for the network trained with the soft-label representation vs the network trained with the distogram representation. Each target was predicted with a single MSA subsample (*N*_eff_ = 10) and 10 randomly subsampled crosslink sets. The sulfo-SDA crosslinks were represented as a uniformly distributed distogram. The soft-label representation outperforms the distogram representation on average by 5%. Source data

In AlphaLink, crosslinks can act as anchors in the prediction itself, since the sparsity of crosslinks is compensated with co-evolutionary information that fills in and extrapolates the missing information. This also enables the software to use co-evolutionary information to perform noise rejection on experimental data. AlphaLink provides a framework for training AlphaFold2-style predictors with a number of data sources providing contacts and/or distance restraints, such as mutagenesis, nuclear magnetic resonance restraints, fluorescence resonance energy transfer and crosslinking MS performed with different crosslinkers. As a test, we fine-tuned the network with simulated sulfo-SDA crosslinks^9^ and could successfully predict our test set (Extended Data Fig. 8).

**Extended Data Fig. 9:**
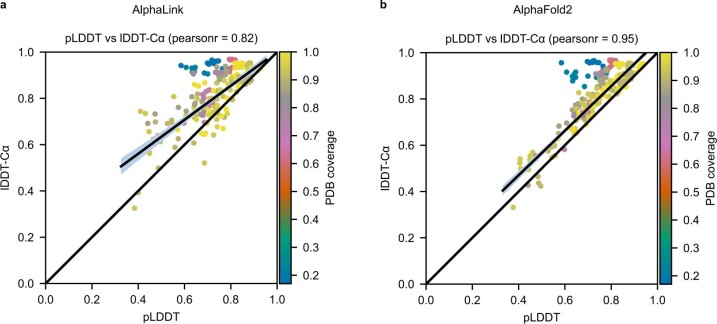
AlphaLink and AlphaFold2 pLDDT correlation. a- We show the calibration of the predicted lDDT-Cα score (pLDDT) for AlphaLink on *N* = 220 predictions of the E. coli membrane fraction dataset. On predictions that are at least 80% covered by the crystal structure, the correlation is 0.82. The true lDDT-Cα score is generally underestimated, meaning that the pLDDT-score is a conservative estimate. b- We show the calibration of the predicted lDDT-Cα score (pLDDT) for AlphaFold2 on N = 220 predictions of the E. coli membrane fraction dataset. On predictions that are at least 80% covered by the crystal structure, the correlation is 0.95.The shaded area corresponds to the 95% confidence interval. Line shows the linear fit. Source data

We validate AlphaLink against CASP14/CAMEO targets that were not part of AlphaLink or AlphaFold2 training using synthetic data, and *E. coli* membrane proteins using in-cell photo-L crosslinking MS data. The crosslinking MS data enabled the systematic testing of predictions of 31 proteins against crystal structures with experimental information. While the gains observed on these targets are more modest than with the CASP14 and CAMEO set, these proteins have known structures that were part of the training set of AlphaFold2. This makes them inherently easier for AlphaFold2 to predict, as a result of data leakage. We show that AlphaLink accurately estimates model confidence with various metrics (predicted lDDT-CA score (pLDDT), predicted TM-score (pTM) and predicted aligned error (PAE)), providing the user with valuable information on what conclusions may be drawn from a particular structure prediction, in a manner comparable to AlphaFold2 (Extended Data Figs. 3, 4 and 9). This is a considerable improvement over the performance of crosslinks in CASP13, where crosslinks were included as information in the data-assisted category and led to a decrease in prediction quality^30^.

Beyond improving predictions on challenging targets, simulated here by low *N*_eff_, AlphaLink opens up the investigation of multiple conformational states by a combination of protein structure prediction and experimental information. This enables the structural characterization of cellular processes in defined biological conditions and may eventually be used to design binders and inhibitors to target specific protein states. Unlike other methods that rely on manipulation of the MSA^25,31,32^, AlphaLink uses experimental information to drive the prediction of multiple conformational states. Because the algorithm weighs experimental evidence against evolutionary information, the nature and size of the MSA plays a role in driving the prediction. Thus, a high *N*_eff_ can ‘overpower’ experimental evidence. In this regard, subsampling the MSA is a way to tune down the weight of the MSA. In the analyses of KaiB and selecase, AlphaLink can be run with multiple MSA subsamplings or even combined with sequence clustering^25^ to characterize the full range of conformations for given combinations of experimental and MSA evidence. Intriguingly, for both KaiB and Cdk2, running AlphaLink with crosslinks from mixtures of conformers led to predictions coinciding with one state at a low *N*_eff_, then predictions in between and finally another state at high *N*_eff_. In the case of selecase, sequence clustering did not produce the alternative conformation at all^25^, while AlphaLink could produce the alternative conformation in the absence of MSA information.

Taken together, our results show that AlphaLink successfully leverages experimental restraints via deep learning to improve protein structure prediction. We present a workflow based on photo-AA crosslinking MS, which provides contact-like distance information, and obtain the first large-scale photo-AA crosslinking MS dataset inside cells. We then implement photo-AA-based protein structure prediction in AlphaLink. Our method leverages a list of generic contacts, represented as explicit distance restraints or as distograms, to guide the OpenFold pipeline towards structures consistent with the experimental data. The workflow outlined here thus provides a general framework for the hybrid experiment-assisted AI prediction of protein structure, investigating the structure–function relationship of proteins directly in situ without any genetic manipulation.

## Methods

### Crosslink simulation

We considered several representations for photo-crosslinking MS-derived contacts (Supplementary Table 2), and ultimately decided to train with 10 Å Cα–Cα contacts, since it agrees well with the mean distances observed in experimental data (Fig. 3b), closely resembles previous definitions of contact restraints^36,37^ and represents experimentally observed distance distributions more accurately than simulating crosslinks from leucine Cδ1 to the nearest nonhydrogen atom.

We simulate crosslinks by taking all residue pairs where one residue is a leucine or lysine and the other residue has an atom that is within 10 Å of the Cα atom of leucine/lysine. We consider only crosslinks that are not within the same or consecutive tryptic peptides. The links are randomly subsampled to 10% to match the expected coverage of the real data. We further add 5% of stochastic noise to match the expected FDR. During training, we always simulate at least one incorrect crosslink. The FDR can therefore be much higher. The link-level FDR is simulated by shuffling the crosslinks and counting the number of incorrect links observed so far. The minimum FDR is 5%. Crosslink statistics for the CASP14/CAMEO set can be found in Supplementary Tables 3–5.

### Distogram sampling

We sample distograms on-the-fly during training to condition the network. There are three different types of distogram: first, a uniformly distributed distogram that represents contact information; second, a distogram based on the expected distances for a specific distance bin; third, a distogram based on the expected distances for photo-L and photo-K crosslinks (10 Å). For all distograms the probabilities sum to 1-FDR below the chosen bin and to FDR beyond the chosen bin.

### Integration of crosslinks

We add a crosslink embedding layer to OpenFold by specifying an additional linear layer that maps the soft-label contact map, or in case of the distogram network, the distograms into the 128-dimensional *z*-space present in the AlphaFold2/OpenFold architecture. The projection is added to the pair representation (*z*). In addition, we learn a group embedding layer to indicate groups of possibly ambiguous crosslinks, which enables us to deal with restraints with positional ambiguity. The group embedding is also added to the pair representation.

### MSA subsampling

For refining the network, we subsample the MSAs to a specific *N*_eff_. The *N*_eff_ corresponds to the number of nonredundant sequences in a MSA below a specific sequence identity. We set the sequence identity to 80%. We subsample the MSAs in training in each epoch to a random *N*_eff_ between 1 and 25, generating a uniform distribution of *N*_eff_ values across all targets. In subsampling, the MSA is shuffled and sequences are added until the desired *N*_eff_ is reached.

### Conformer experiment

For KaiB (Q79V61) and selecase (Q58610) we produced a total of 200 predictions that contained 100 simulated crosslinks (FDR 5%) and a set of 4–7 crosslinks unique to each conformation. The mixture crosslinking set is subsampled from both sets to ensure similar coverage. The unique crosslinks for each conformation are added on top.

### Fine-tuning of AlphaFold2

**Extended Data Fig. 10:**
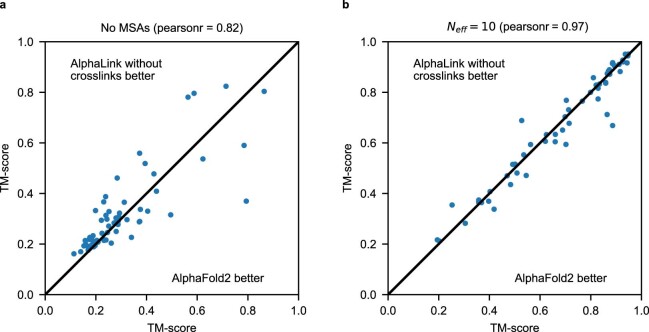
Refining on low *N*_eff_ targets doesn’t change results substantially. a- Performance improvements we observed are due to adding crosslinking information, not additional fine-tuning of the AlphaFold2 weights on low *N*_eff_ targets. There are few outliers on both sides. The performance is virtually identical for targets without MSAs (TM-score average: AlphaLink = 0.322, AlphaFold2 = 0.308, Z-statistic = −0.072). b- Performance improvements we observed are due to adding crosslinking information, not additional fine-tuning of the AlphaFold2 weights on low Neff targets. There are few outliers on both sides. The performance is virtually identical for *N*_eff_ = 10 (TM-score average: AlphaLink = 0.701, AlphaFold2 = 0.702, Z-statistic = 0.033). Points show the mean (*N* = 10) over 10 MSA subsamples. Source data

To avoid training OpenFold from scratch, we start with the AlphaFold2 2.0 (https://github.com/deepmind/alphafold/releases/tag/v2.0.0) weights provided by Deepmind and refine the network on 13,000 proteins from the trRosetta^38^ training set with simulated photo-AA crosslinking data. We use OpenFold v0.1.0 (based on GitHub from January 2022: commit 894905b9da941ed10e797c5ba15af75692cee1b4). To encourage the network to use the crosslinking data, we subsample the MSA to a *N*_eff_ between 1 and 25 (uniformly). MSAs were generated with the reduced database setting. We train and test with model_5_ptm, which does not use any template information. Fine-tuning specifically on low *N*_eff_ targets does not substantially change the performance of AlphaLink. We predicted the same structures without crosslinks in AlphaLink to verify that fine-tuning the network on low *N*_eff_ targets is not the reason for our improvements (Extended Data Fig. 10).

We follow the refinement training regime outlined in the AlphaFold2 paper, except that, due to memory constraints, we do not expand the MSA cluster size. Since our method is specifically targeting proteins with few MSAs, this is not a problem. We train for five epochs on five GPUs, which takes roughly 5 days.

### Evaluation set up

For the baseline, we use OpenFold with the original AlphaFold2 weights provided by Deepmind. The creators of OpenFold verified that the implementation produces identical results. To assess AlphaLink and Openfold performance, we perform predictions with the model_5_ptm setting, which does not include templates and predicts the TM score as an auxiliary loss.

To ensure comparability, we make predictions deterministic. We disable masking out parts of the MSA input. Especially on targets with small MSAs, masking out parts of the input leads to big variances between runs, since it affects the *N*_eff_. Here reconstruction can increase the *N*_eff_. We use a fixed set of ten subsampled MSAs and a fixed set of ten subsampled crosslinks. Normally, MSAs will be subsampled on-the-fly to 128 sequences. The rest is aggregated with the ExtraMSAStack. We cap our MSA size at 128 for the subsampled MSAs to remove variance. Since we mostly evaluate on MSAs with *N*_eff_ = 10, where the MSA size is far below 128, we seldom reach this limit in practice.

If not denoted otherwise, the results we will show use the soft-label representation, which is trained for the particular crosslinker type and performs slightly better.

Our main comparison metric is the template modeling score (TM score), which measures the similarity between two protein structures. The TM score is calibrated in a way that structures with a TM score above 0.5 generally assume the same fold. A TM score of 1.0 signifies a perfect match. TM scores <0.2 correspond to random structures.

We use the same databases as Deepmind to recreate the CASP14 settings: UniRef90: v2020_01, MGnify: v2018_12, Uniclust30: v2018_08, BFD: only version available, PDB: downloaded 14 May 2020, PDB70: 13 May 2020. The MSAs are generated with the reduced database setting. We evaluate the CASP14 targets on the full sequence, not splitting them into domains. We evaluate on 45 CAMEO targets that were released after AlphaFold2. We consider only CAMEO targets where AlphaFold2 does not exceed a TM score of 0.8. We used SMART with pFam annotation^39^ to divide the CAMEO targets into single/multidomain, ignoring low-complexity regions.

For predictions of conformational states of Cdk2, potential photo-L and photo-K crosslink sites were derived from structures of inhibited (1h01) (ref. ^34^) and cyclin-A bound states (2bpm) (ref. ^35^). Separate AlphaLink predictions were submitted with either dataset at *N*_eff_ = 10. AlphaFold2 predictions were performed at *N*_eff_ = 10 with the model_5_ptm setting. For Extended Data Fig. 5a, AlphaFold2 predictions were carried out with full MSA size and default model settings (five random seeds per model, five models predicted).

### Photo-L crosslinking of *E. coli* cells

For optimization of photo-L concentration in the medium, *E. coli* K12 were grown in LB medium overnight at 37 °C. The cultures were diluted (1:100) into M9 minimal medium containing 0.2% glucose and varying concentrations of photo-L (0, 5, 25, 250, 500, 750, 1,000 and 2,000 µM photo-L) in 96-well plates in a Microplate reader Infinite M200 Tecan. Three colonies were used per condition. Cell growth was monitored via OD_600_.

For crosslinking MS experiments, *E. coli* were grown with 0.75 mM photo-L for 22 h at 37 °C in 100 ml of M9 minimal medium. Thirty million cells were then pelleted for 15 min at 4,000*g*. The pellet was resuspended in fresh M9 minimal medium to a concentration of 1 million cells ml^−1^. UV crosslinking was then performed by exposing the cell suspension for 20 min on ice in a CL-1000L crosslinking device (UVP). Cells were then pelleted again, washed with PBS buffer and snap frozen.

### Membrane enrichment

Cells were resuspended in 20 mM Tris, pH 7.4 and lysed by four freeze–thaw cycles followed by sonication. Larger cell debris was removed through centrifugation at 2,000*g* for 20 min. The supernatant was then cleared centrifuged at 16,000*g* for 20 min at 4 °C. The pellet was washed with 20 mM Tris pH 7.6, 1 M NaCl.

### Proteome digestion and peptide fractionation

The pellets were solubilized in PBS buffer and subsequently mixed with NuPage LDS sample buffer (Life Technologes) and run into a 4–12% Bis-Tris SDS–PAGE gel (Life Technologies). Gels were stained using Imperial Protein Stain (Thermo Scientific), and the whole proteome was cut out and prepared for in-gel digestion. Proteins were reduced with 10 mM dithiothreitol (Sigma Aldrich) for 30 min at 37 °C, followed by alkylation with 55 mM iodoacetamide (Sigma Aldrich) for 20 min at room temperature in the dark. Gel pieces were incubated with 13 ng μl^−1^ trypsin (Pierce, Thermo Fisher Scientific) at 37 °C for 16 h in 10 mM ammonium bicarbonate, 10% acetonitrile (ACN). The samples were cleaned up using Sep-Pak C18 cartridges (Waters) before strong cation exchange chromatography.

The peptides were resuspended in SCX loading buffer (10 mM KH_2_PO_4_ and 30% ACN) and separated on a polysulfoethyl A column (PolyLC, PolySulfoethyl A 100 × 2.1 mm^2^, 3 µm) using SCX elution buffer (10 mM KH_2_PO_4_, 30% ACN and 1 M KCl). Separation of peptides was accomplished using a nonlinear gradient with running buffer B (30% ACN, 1 M KCl and 10 mM KH_2_PO_4_, pH 3.0), as described^40^. Fractions of 200 µl each were collected over the elution window (approximately 18 column volumes). Collected fractions of interest from five runs were pooled, desalted using Stage-Tips and stored at −20 °C.

Crosslinked peptides in each SCX fraction (labeled fractions 16–22 in the JPOST deposition) were subsequently enriched by size-exclusion chromatography using a Superdex Peptide 3.2/300 column (GE Healthcare). The mobile phase consisted of 30% (v/v) ACN and 0.1% trifluoroacetic acid, running at a flow rate of 10 μl min^−1^. The earliest five peptide-containing fractions (50 μl each, labeled SEC6–10) were collected and dried in a vacuum concentrator. Whenever amounts were insufficient for liquid chromatography (LC)–MS analysis, adjacent fractions were pooled.

### LC–MS acquisition of photo-L crosslinked *E. coli* membranes

Acquisition of crosslinked peptide spectra was performed on a Fusion Lumos Tribrid Mass Spectrometer (Thermo Fisher Scientific) connected to an Ultimate 3000 UHPLC system (Dionex) operating with XCalibur 4.4 and Tune 3.4. Chromatography was performed with mobile 0.1% (v/v) formic acid as mobile phase A, and 80% (v/v) ACN, 0.1% (v/v) formic acid as mobile phase B. The samples were dissolved in 1.6% ACN (Honeywell Fluka), 0.1% formic acid (Honeywell Fluka) and separated on an Easy-Spray column (C-18, 50 cm, 75 µm internal diameter, 2 µm particle size, 100 Å pore size) running with 300 nl min^−1^ flow rate using optimized gradients for each offline fraction (ranging from 2% B to 55% B over 62.5, 92.5 or 152.5 min, then to 55% in 2.5 min and to 95% in 2.5 min).

The MS data were acquired in data-dependent mode using the top-speed setting with a 3 s cycle time. For every cycle, the full-scan mass spectrum was recorded in the Orbitrap at a resolution of 120,000 in the range of 400 to 1,450 *m*/*z*. Ions with a precursor charge state between +3 and +7 were isolated and fragmented with a decision tree strategy^41^. Higher-energy collisional dissociation energies optimized for mass and charge of a precursor species were applied^41^. The fragmentation spectra were then recorded in the Orbitrap with a resolution of 50,000. Dynamic exclusion was enabled with single repeat count and 60 s exclusion duration.

### Identification and statistical validation of crosslinked peptides

LC-MS raw data were searched against the *E. coli* K12 proteome (download from UniProt February 2020) using MaxQuant 1.6.17 (ref. ^42^) (Supplementary Data 3). The top 1,200 proteins by iBAQ were used as the database for the crosslink search. For the crosslink search, raw data were processed using MSconvert 3.0.22 (ref. ^43^) to recalibrate precursor masses and convert to mgf format. An open modification search with MSfragger 3.4 (ref. ^44^) was used to quantify modifications in the sample. The peak files were then searched with xiSEARCH 1.7.6.4 (ref. ^45^) with the following settings: MS1 tolerance: 3 ppm; MS2 tolerance: 5 ppm, allowing up to two missing monoisotopic peaks and three missed tryptic cleavages. Cysteine carbamidomethylation was defined as a fixed modification. Oxidation of methionine, deamidation of asparagine and methylation of glutamic acid were defined as variable modifications. –CH_3_SOH/–H_2_O/–NH_3_ were defined as losses. In the crosslink search, the photo-L crosslinker was defined as follows: the linkage mass was set to −16.0313 Da and the specificity set to leucine to any amino acid. Variable modifications on leucine to account for photo-L reactions with water (1.97926 Da) or within a peptide (−16.0313) were also defined. Noncovalent gas-phase associations were included in the search^46^.

The spectral matches were filtered before FDR estimation to crosslinked peptide matches with a minimum of three fragments matched per peptide. Results were then filtered to 5% crosslink-level FDR in xiFDR 2.1.5.5 (ref. ^45^) with the boosting feature for error thresholding at lower levels enabled. The minimum peptide length was set to 6. Consecutive peptides were removed from the analysis. The resulting residue pairs are in Supplementary Data 2.

### Reporting summary

Further information on research design is available in the Nature Portfolio Reporting Summary linked to this article.

## Online content

Any methods, additional references, Nature Portfolio reporting summaries, source data, extended data, supplementary information, acknowledgements, peer review information; details of author contributions and competing interests; and statements of data and code availability are available at 10.1038/s41587-023-01704-z.

### Supplementary information

Supplementary Information Extended Data Figs. 1–10 and supplementary data legends.

Reporting Summary

Supplementary Tables This excel file contains Supplementary Tables 1–5 in separate sheets. **Supplementary Table 1.** Restraints for inhibited and cyclin-bound Cdk2 predictions. **Supplementary Table 2.** Simulated crosslink data used for training of AlphaLink. **Supplementary Table 3.** Crosslink simulation for 60 CASP14 targets. **Supplementary Table 4.** Crosslink simulation for 45 CAMEO targets. **Supplementary Table 5.** Crosslink simulation for 50 low *N* CAMEO targets.

Supplementary Data 1 AlphaLink performance benchmark on CAMEO and CASP14 targets.

Supplementary Data 2 *E. coli* membrane fraction photo-L crosslinks.

Supplementary Data 3 Protein abundances for crosslink search database.

Supplementary Data 4 AlphaLink performance benchmark on the *E. coli* membrane fraction.

### Source data

Source Data Fig. 2 AlphaLink performance benchmark on CAMEO and CASP14 targets.

Source Data Fig. 3 Distance distributions for in-cell crosslinks mapped on experimental structures.

Source Data Fig. 4 AlphaLink performance benchmark on the *E. coli* membrane fraction.

Source Data Extended Data Fig./Table 1 AlphaLink performance on CAMEO and CASP14 benchmark dataset and performance with distogram representation.

Source Data Extended Data Fig./Table 2 Raw data for growth curves in panel a. Panel b based on Supplementary Data 2.

Source Data Extended Data Fig./Table 3 Raw data for TM-score–pTM correlation on the outer membrane proteins (AlphaFold2).

Source Data Extended Data Fig./Table 4 AlphaLink pTM scores for outer membrane proteins, including proteins that have no crystal structure.

Source Data Extended Data Fig./Table 5 Raw data for the conformer experiment. AlphaLink predictions for KaiB and selecase.

Source Data Extended Data Fig./Table 6 AlphaLink and OmegaFold TM scores for CAPS14 targets.

Source Data Extended Data Fig./Table 7 Prediction time (s) for AlphaLink and AlphaFold2 for CASP14 targets.

Source Data Extended Data Fig./Table 8 AlphaLink and AlphaFold2 TM scores on CASP14 targets (*N*_eff_ = 10). AlphaLink predictions were made with sulfo-SDA (distogram and soft-label representation).

Source Data Extended Data Fig./Table 9 lDDT-CA and pLDDT for AlphaFold2 and AlphaLink on the outer membrane proteins with crystal structures.

Source Data Extended Data Fig./Table 10 AlphaFold2 and AlphaLink TM-scores on CASP14 targets. AlphaLink predictions without crosslinks.

## Data Availability

Crosslinking MS data are deposited in ProteomeXChange JPOST^47^ with accession code JPST001851. Models and corresponding MSA and simulated crosslinking data have been deposited on ModelArchive^48^ with accession code ma-rap-alink. AlphaLink models based on experimental crosslinks have been deposited as integrative/hybrid models in PDB-Dev^49^ with accession codes PDBDEV_00000165 to PDBDEV_00000198 (group ID PDBDEV_G_1000001). Source data are provided with this paper.

## Code availability

The code for AlphaLink is deposited at https://github.com/lhatsk/AlphaLink.
