# Supplementary material for: Protein structure prediction with in-cell photo-crosslinking mass spectrometry and deep learning
Source: Nat Biotechnol. 2023 Mar 20;41(12):1810–9. doi: 10.1038/s41587-023-01704-z (PMC10713450; doi:10.1038/s41587-023-01704-z)
Supplement: Supplementary file 1 — Extended Data Figs. 1–10 and supplementary data legends. [file 41587_2023_1704_MOESM1_ESM.pdf]

nature biotechnology

Article https://doi.org/10.1038/s41587-023-01704-z

Protein structure prediction with in-cell photo-crosslinking mass spectrometry and deep learning

In the format provided by the authors and unedited

Supplementary data S1: AlphaLink performance benchmark on CAMEO and CASP14 targets.

Supplementary data S2: *E. coli* membrane fraction Photo-L Crosslinks.

Supplementary data S3: Protein abundances for crosslink search database.

Supplementary data S4: AlphaLink performance benchmark on the *E. coli* membrane fraction.

Source Data Fig. 2: AlphaLink performance benchmark on CAMEO and CASP14 targets.

Source Data Fig. 3: distance distributions for in-cell crosslinks mapped on experimental structures.

Source Data Fig. 4: AlphaLink performance benchmark on the *E. coli* membrane fraction.

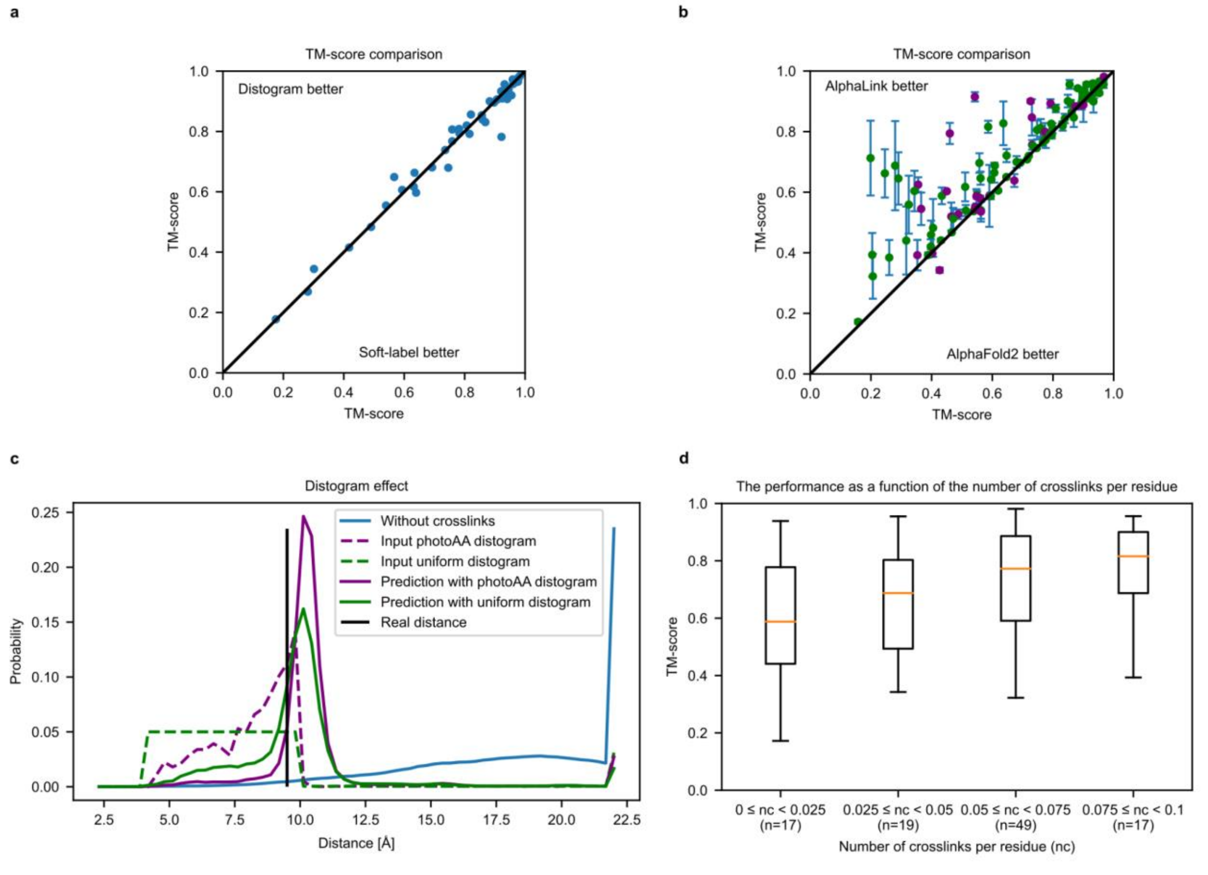

Extended Data Figure 1: Performance on benchmark data set and distogram effect

a- TM-score comparison (N = 10) 49 challenging CAMEO targets for the network trained with the soft-label representation and the network trained with the distogram representation. Each target was predicted with 10 randomly subsampled crosslink sets. Scatter points show the mean. The photo-L crosslinks were represented as a uniformly distributed distogram. The performance is on par. **b-** Performance on 60 CASP14 and 45 CAMEO (N_{eff} = 10). AlphaLink improves the TM-score on average by 15.2%. The error bars represent the 95% confidence interval (N = 10). Points show the mean. Purple highlights multi-domain targets, green highlights single-domain targets. **c-** The effect two different distogram inputs (dashed) have on the distogram AlphaLink predicts (solid) between residues 11 and 103 for T0164. The solid black line designates the real distance. Blue shows the distogram without using crosslinks. The green distogram mimics an upper bound contact restraint, while the purple distogram is the expected distance distribution for simulated photo-L and photo-K crosslinks on the training set. Predicted distogram based on the expected photoAA distance distribution (purple, solid) is more narrow compared to the prediction with the uniform distance distribution (green, solid). Using a distance distribution improves the prediction from TM-score of 0.68 to 0.7. We show the first 64 bins of the input distograms and sum up the probabilities for the rest. In the absence of this restraint, the prediction has a TM-score of 0.28 (N_{eff} = 10). **d-** Performance of AlphaLink as a function of the number of crosslinks per residue (N_{eff} = 10). 2 bins (nc > 0.1) were omitted because they only contained 1 and 2 samples. Performance generally increases with more crosslinks per residue. The line shows the median and the whiskers represent the 1.5x interquartile range.

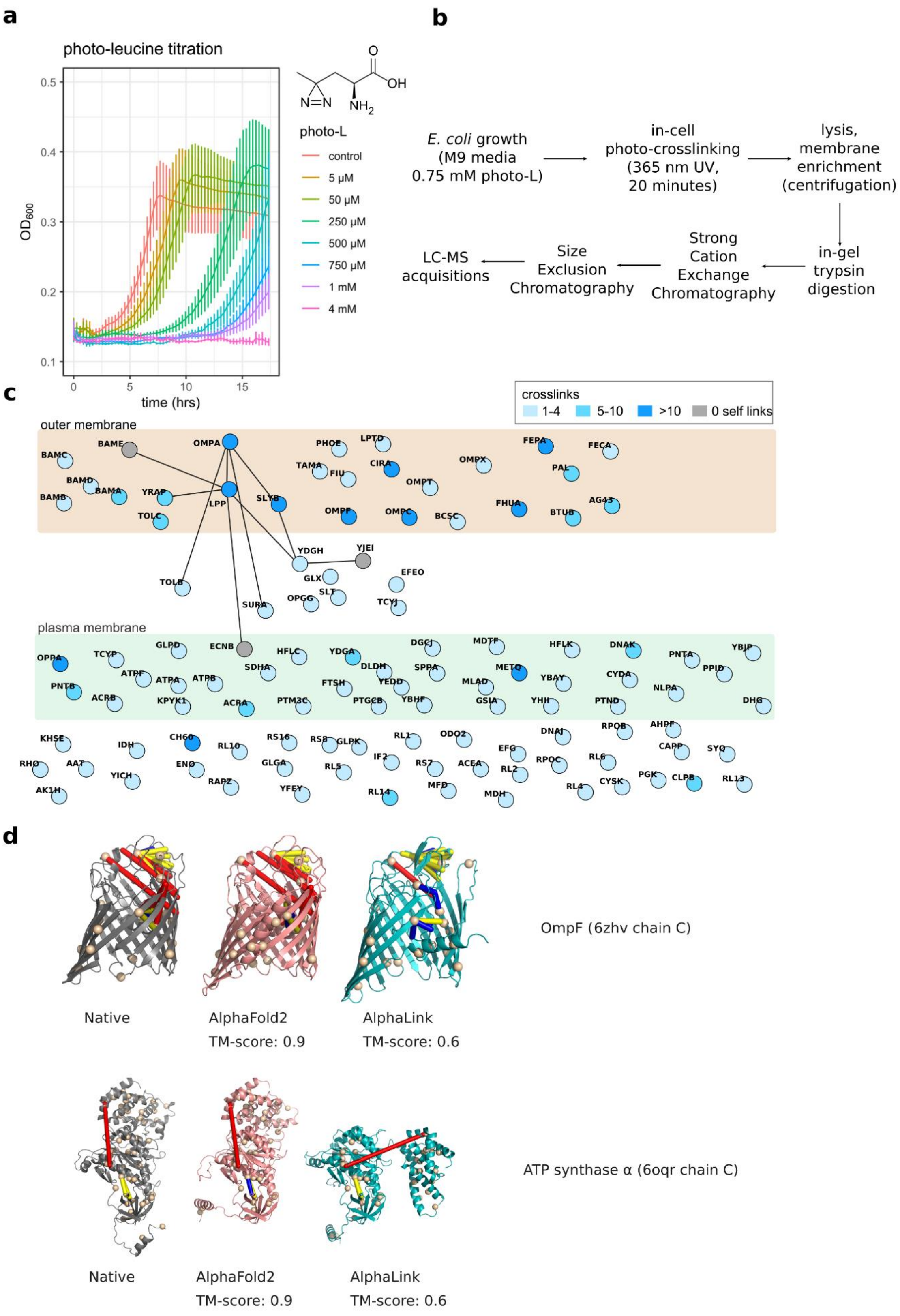
a
photo-leucine titration
OD600
time (hrs)
photo-L
control
5 µM
50 µM
250 µM
500 µM
750 µM
1 mM
4 mM
NH2
OH
O
b
E. coli growth (M9 media 0.75 mM photo-L)
in-cell photo-crosslinking (365 nm UV, 20 minutes)
lysis, membrane enrichment (centrifugation)
in-gel trypsin digestion
Strong Cation Exchange Chromatography
Size Exclusion Chromatography
LC-MS acquisitions
c
crosslinks
1-4
5-10
>10
0 self links
outer membrane
plasma membrane
d
OmpF (6zhv chain C)
Native
AlphaFold2
TM-score: 0.9
AlphaLink
TM-score: 0.6
ATP synthase α (6oqr chain C)
Native
AlphaFold2
TM-score: 0.9
AlphaLink
TM-score: 0.6

Extended Data Figure 2: photo amino acid crosslinking MS workflow and membrane outliers.

a- *E. coli* K12 grown with increasing concentration of photo-L in M9 minimal media and structure of photo-L. Average cell density with standard error across 3 individual colonies is plotted. **b-** Workflow for two-dimensional fractionation of peptides from *E. coli* crosslinked with photo-L in cell. **c-** Nodes represent proteins detected with at least one crosslink, and edges indicate the presence of at least a crosslinked residue pair between proteins. Nodes are coloured according to the number of crosslinks detected in that protein. **d-** Two examples where crosslinks negatively impact the performance in AlphaLink. The crosslink sets contain overlength links (shown in red in the native structure) which cause movements in AlphaLink (e.g., domain movement in ATP synthase - likely a false positive link). OmpF is homo-multimeric, the overlength links might stem from links between different subunits.

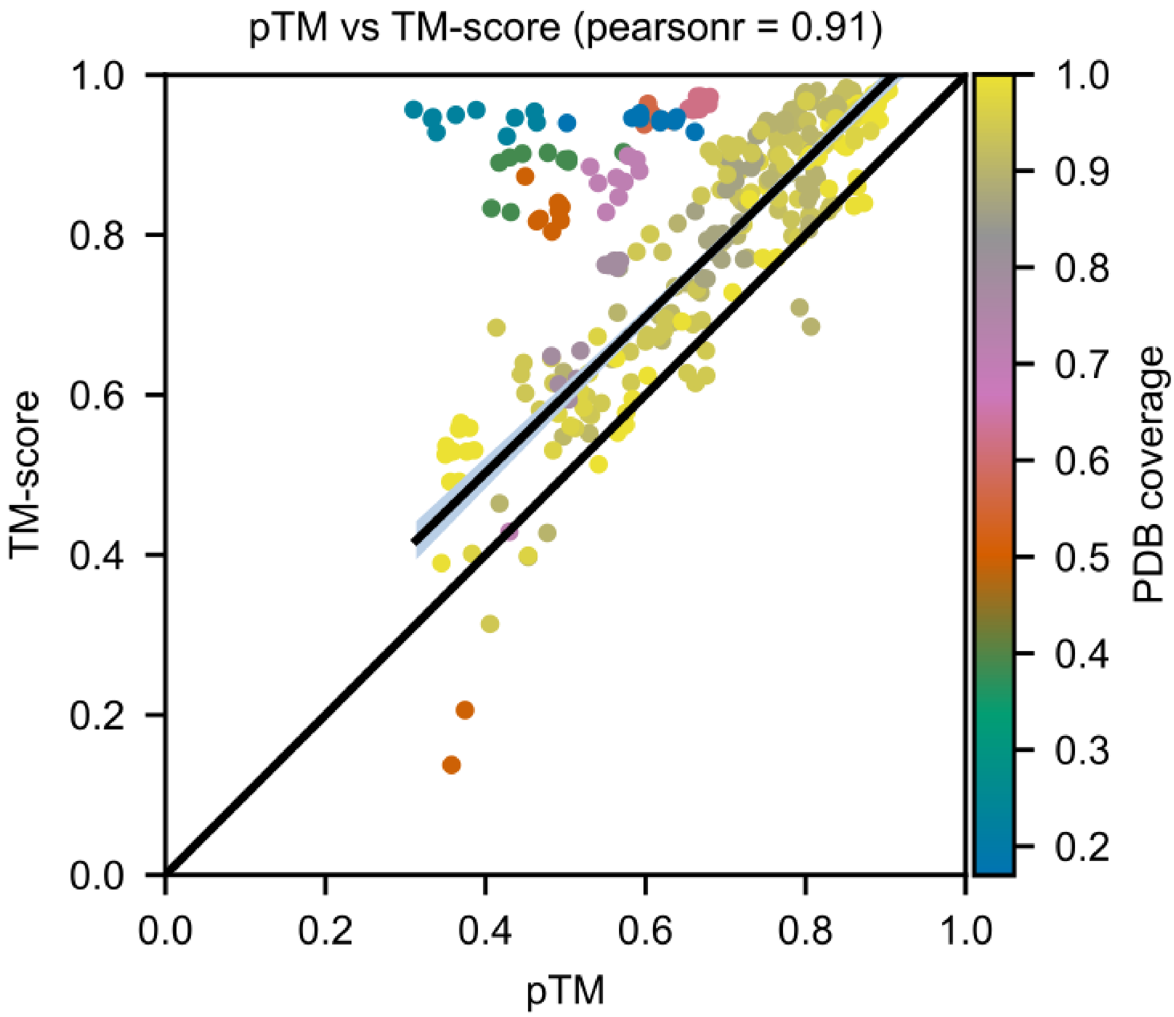

Extended Data Figure 3: AlphaFold2 pTM correlation

Calibration of the predicted TM-score (pTM) on N = 320 predictions of the *E. coli* membrane fraction dataset. On predictions that are at least 80% covered by the crystal structure, the correlation is 0.91. The true TM-score is generally underestimated, meaning that the pTM-score of AlphaFold2 is a conservative estimate. The shaded area corresponds to the 95% confidence interval. Line shows the linear fit.

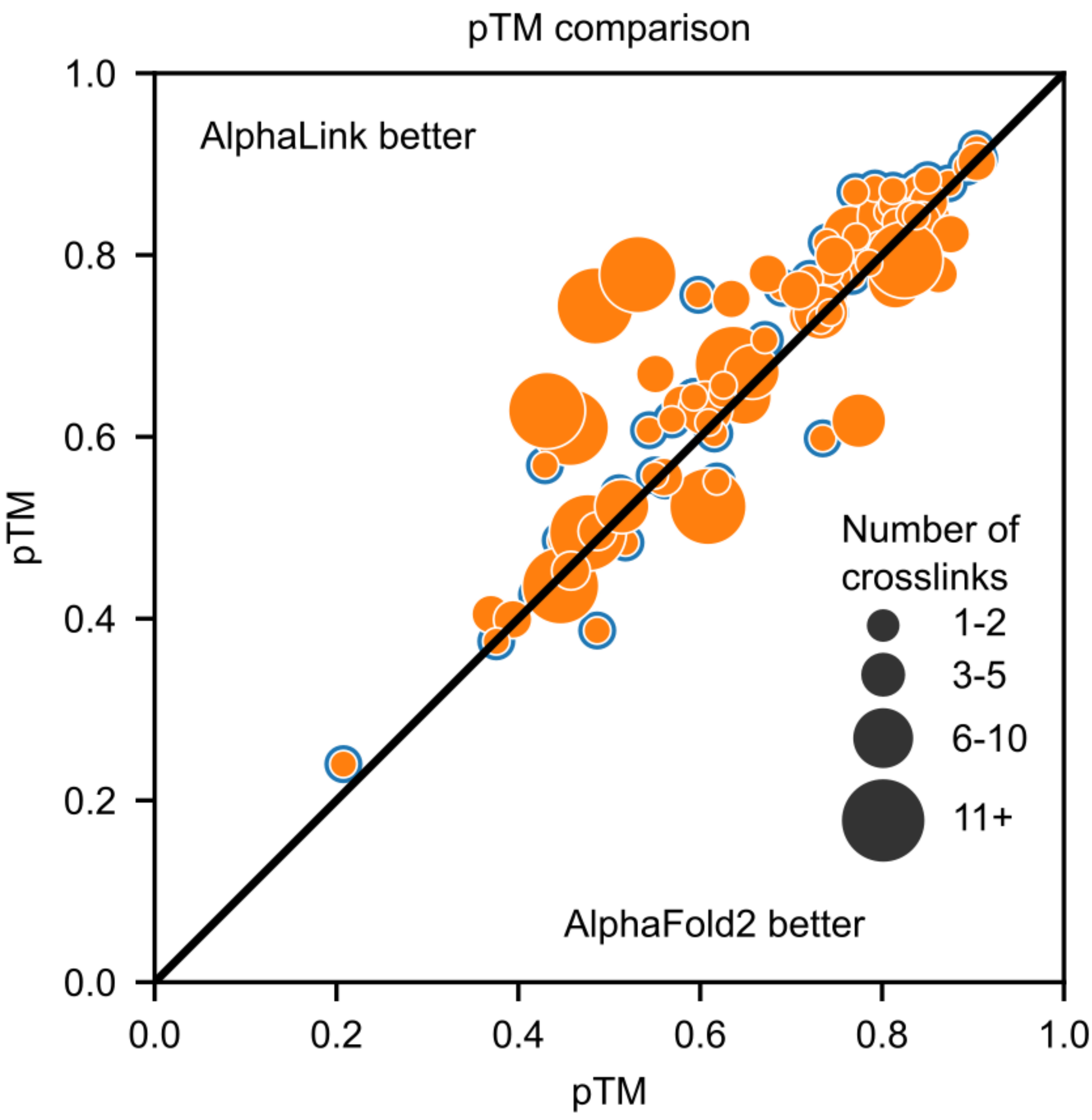

Extended Data Figure 4: pTM comparison on 96 proteins from the *E. coli* membrane fraction dataset

pTM comparison on N = 96 proteins from the *E. coli* membrane fraction dataset. Each points is one protein with one MSA subsample (N_{eff} = 10). The higher pTM of AlphaLink indicates improved structures.

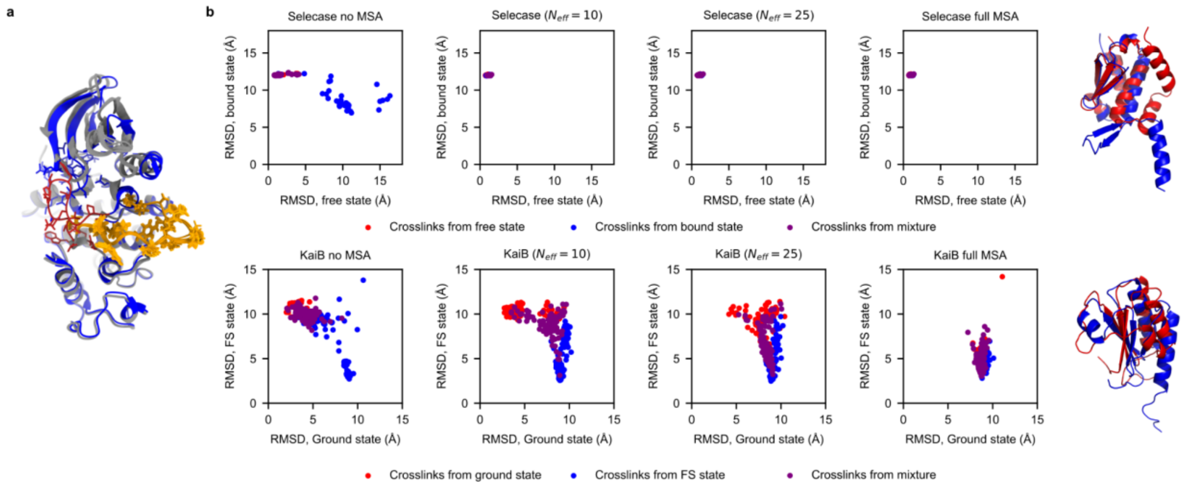

Extended Data Figure 5: Predicting multiple conformations

a- Cdk2 predictions by AlphaFold2.2 with default settings (full MSA, 5 models predicted, 5 random seeds per model), in gray, overlaid with the structure of the inhibited conformation of Cdk2 (blue, pdb 1h01). The T-loop is highlighted in orange in the AlphaFold2 predictions and in red in the inhibited conformation structure. All 25 AlphaFold2 predictions converge on the cyclin A-bound conformation of the T-loop and PSTAIRE helix of Cdk2. **b-** Each points is one prediction with a randomly sampled crosslink (FDR = 5%) set which includes additional links from the highlighted conformation. 100 samples per conformation. For Selecase, AlphaLink always predicts the free state with high accuracy if we use MSAs. Without MSAs, some predictions are driven towards the bound state. For KaiB, AlphaLink is able to predict the ground state with good precision without MSAs. Introducing co-evolutionary information leads to better clustering, although some of the ground state predictions now end up in the average state. This movement proceeds with more co-evolutionary information. With full MSAs, almost all predictions have moved to the fold-switched state.

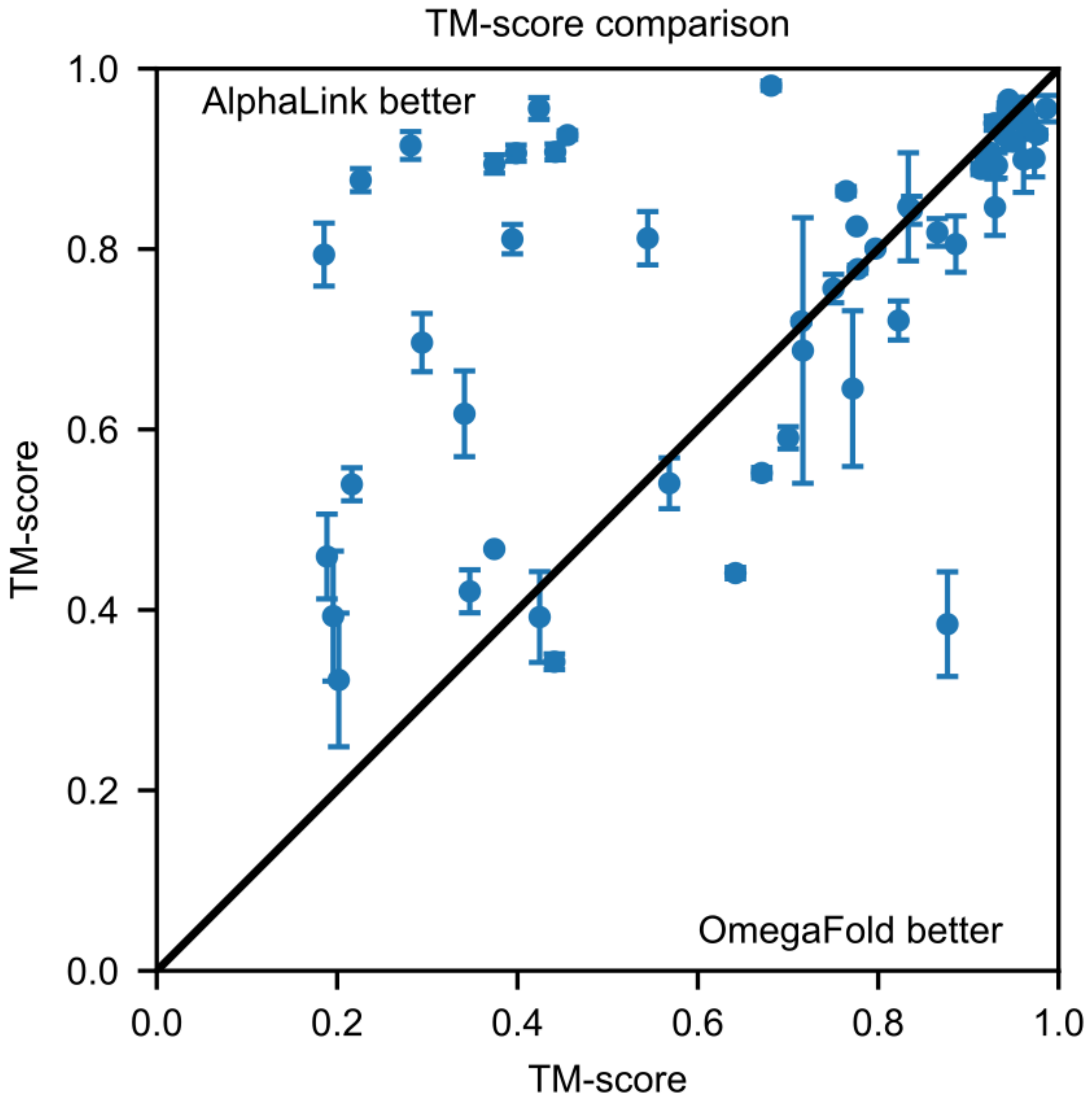

Extended Data Figure 6: AlphaLink vs OmegaFold

AlphaLink vs OmegaFold performance on 58 CASP14 targets with $N_{eff} = 10$ (test set) with simulated photo-L crosslinks. AlphaLink improves the TM-score on average by 36.7% ± 76.6. AlphaLink improves 10 additional targets past a TM-score > 0.5. Shown here is the mean and 95% confidence interval (N= 10) for AlphaLink and the corresponding performance of OmegaFold.

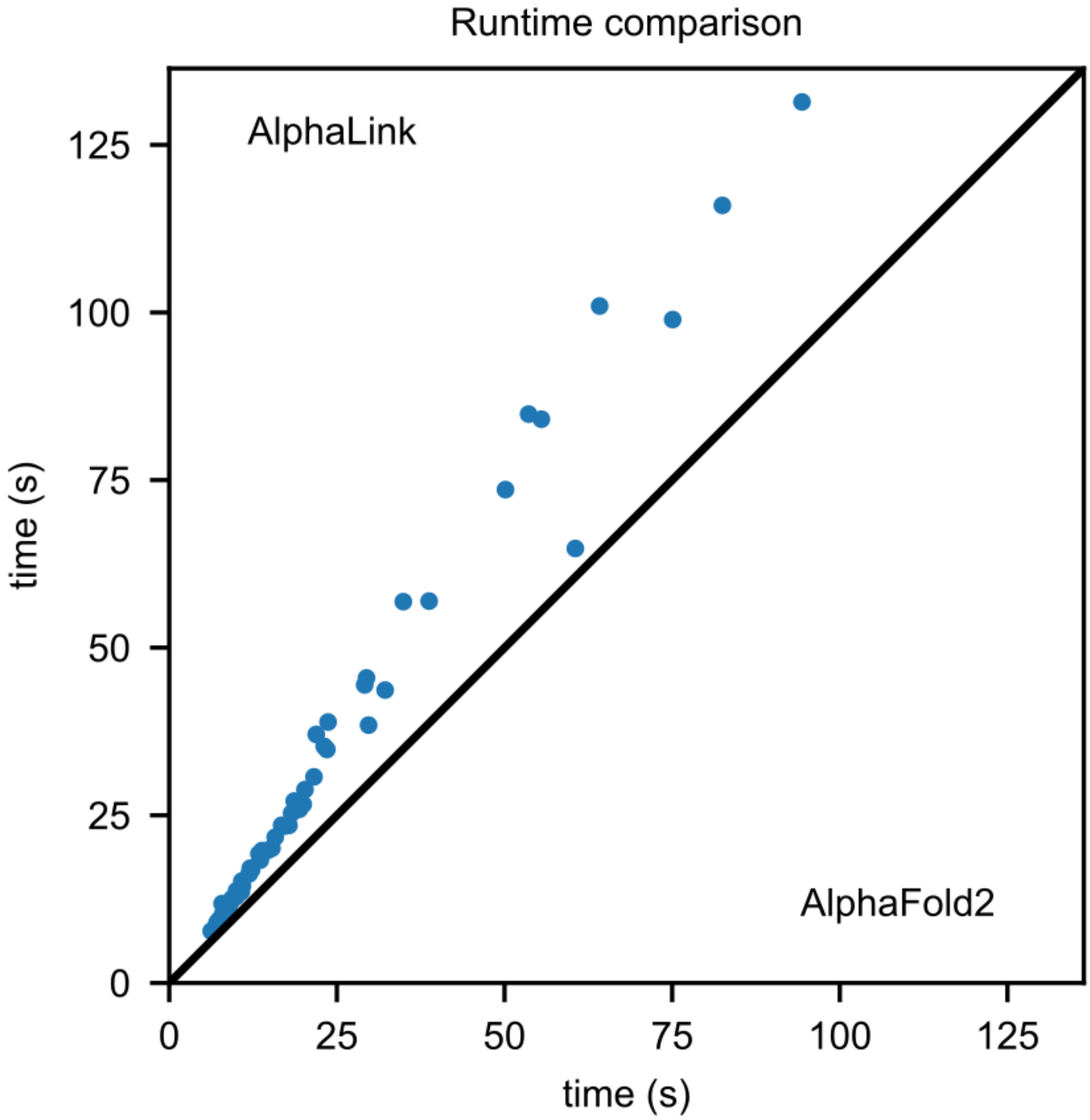

Extended Data Figure 7: AlphaLink vs AlphaFold2 timings

Tested on N = 60 CASP14 targets with N_{eff} = 10. We ran inference, including relaxation on pre-computed features with model_5_ptm. Timed on a node with a single Nvidia A100 80GB GPU and 2 Intel XEON Gold 5118 CPUs (2x24 cores) with 2.3GHz. Mean running time (s) for AlphaLink is 31.7 ± 7.4 s and 22.14 ± 5.16 s for AlphaFold2.

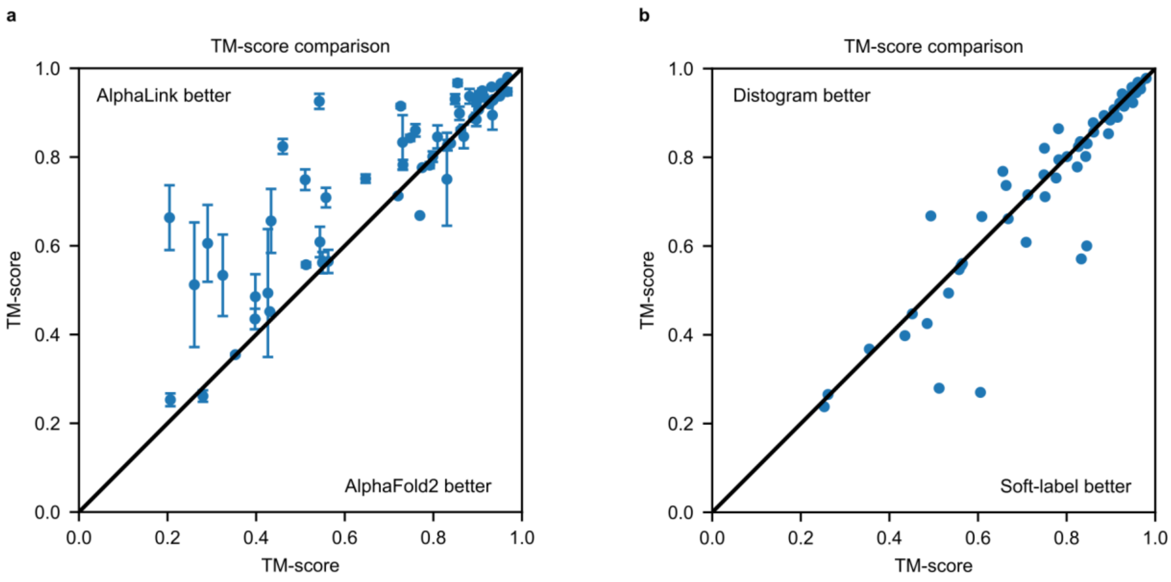

Extended Data Figure 8: AlphaLink with soluble crosslinker on CASP14

a- AlphaLink vs AlphaFold2 performance on 59 CASP14 targets with $N_{eff} = 10$ (test set) with simulated SDA crosslinks. AlphaLink improves the TM-score on average by 16.4% ± 9.7. AlphaLink predicts 5 additional targets with a TM-score > 0.5. Shown here is the mean and 95% confidence interval (N = 10) for AlphaLink and the corresponding performance of AlphaFold2. **b-** We compare the mean TM-score (N = 10) per target on 59 CASP14 targets for the network trained with the soft-label representation vs the network trained with the distogram representation. Each target was predicted with a single MSA subsample ($N_{eff} = 10$) and 10 randomly subsampled crosslink sets. The sulfo-SDA crosslinks were represented as a uniformly distributed distogram. The soft-label representation outperforms the distogram representation on average by 5%.

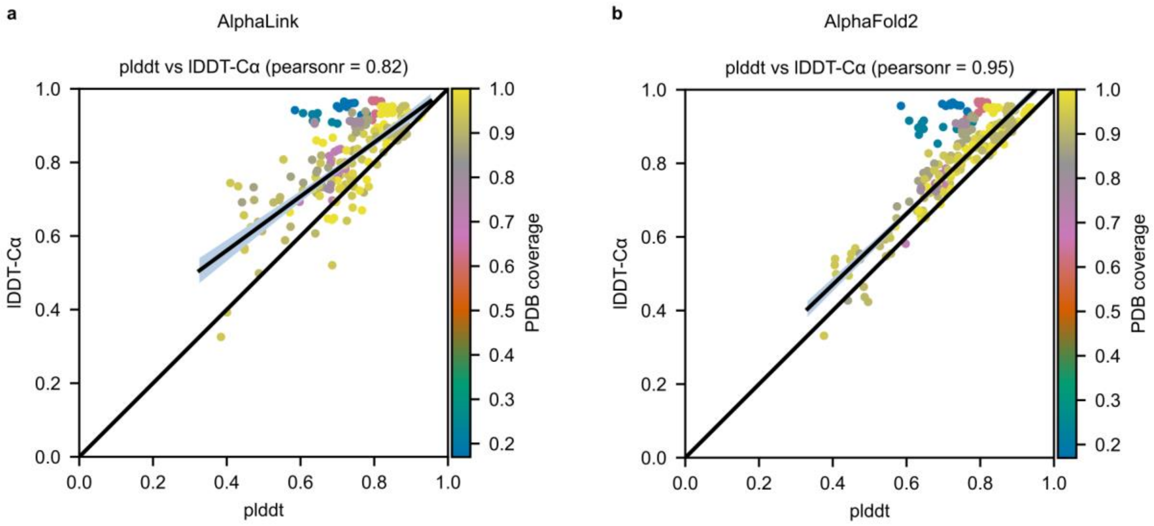

Extended Data Figure 9: AlphaLink and AlphaFold2 pLDDT correlation

We show the calibration of the predicted lDDT-Cα score (pLDDT) on N = 220 predictions of the *E. coli* membrane fraction dataset. On predictions that are at least 80% covered by the crystal structure, the correlation is 0.82 for AlphaLink and 0.95 for AlphaFold2. The true lDDT-Cα score is generally underestimated, meaning that the pLDDT-score is a conservative estimate. The shaded area corresponds to the 95% confidence interval. Line shows the linear fit.

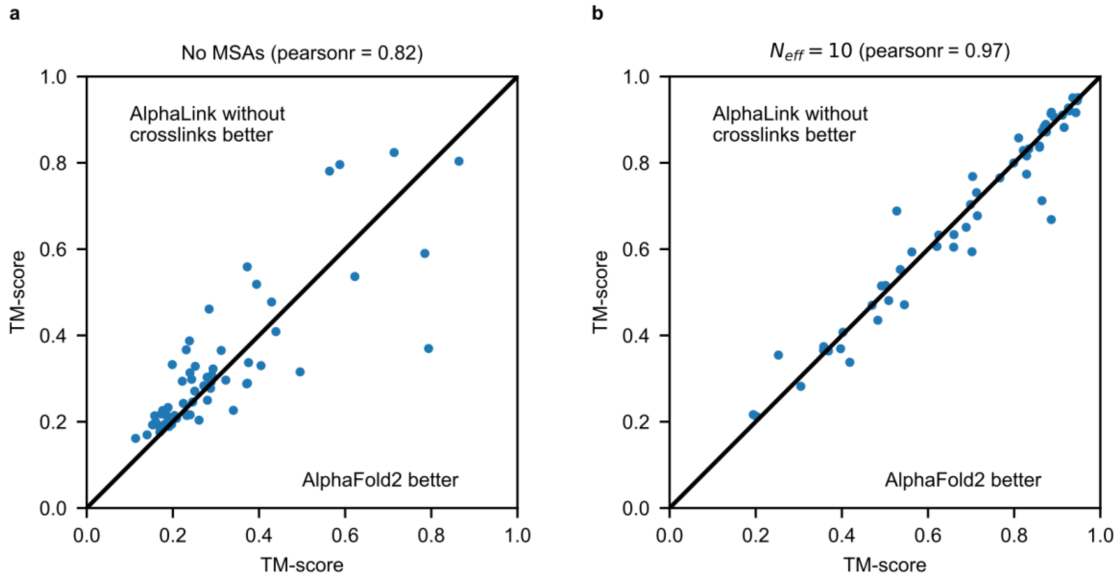

Extended Data Figure 10: Refining on low N_{eff} targets doesn't change results significantly

Performance improvements we observed are due to adding crosslinking information, not additional refining of the AlphaFold2 weights on low N_{eff} targets. There are few outliers on both sides. The performance is virtually identical for N_{eff} = 10 (TM-score average: AlphaLink = 0.701, AlphaFold2 = 0.702, Z-statistic = 0.033) and no MSAs (TM-score average: AlphaLink = 0.322, AlphaFold2 = 0.308, Z-statistic = -0.072). For N_{eff} = 10 points show the mean (N = 10) over 10 MSA subsamples.
